# The Semantic Units Framework, a technology-agnostic representational approach to FAIR and CLEAR knowledge infrastructures

**DOI:** 10.1038/s41597-026-07588-3

**Published:** 2026-06-24

**Authors:** Lars Vogt

**Affiliations:** Leibniz Institute for the Analysis of Biodiversity Change (LIB), Museum of Nature Hamburg, Martin-Luther-King Platz 3, 20146 Hamburg, Germany

**Keywords:** Research data, Research management

## Abstract

This manuscript introduces the Semantic Units Framework, a technology-agnostic representational approach to semantic modularization in which statements and compound meaning structures are treated as first-class semantic units with explicit boundaries, identity, and epistemic status. Motivated by recurring limitations of triple-centric and OWL-based knowledge graphs, the framework shifts the focus from isolated triples to coherent units of meaning that can be referenced, contextualized, and composed. To support scientific generalization beyond individual facts, the framework introduces instance-quantified resource categories (*some-instance*, *most-instances*, *every-instance*, *all-instances*), enabling explicit representation of existential, prototypical, and universal claims, as well as their negations and contextual qualifications. Combined with semantic units, this approach supports the representation of higher-order statements, epistemic stances, and structured scientific argumentation within a unified semantic model. Rather than proposing a new graph technology, the Semantic Units Framework provides a reusable conceptual foundation that can be realized across existing data and knowledge representation technologies and supports both machine-actionable reasoning and human-interpretable knowledge representation.

## Introduction

As the volume and diversity of data and knowledge generated in scientific and societal contexts continue to grow rapidly^1–3^, research data infrastructures are increasingly expected to adhere to the FAIR Guiding Principles^4^. FAIR aims to ensure that data and metadata are Findable, Accessible, Interoperable, and Reusable, thereby supporting the automation of data management processes and enabling large-scale computational use. More recently, the CLEAR Principle has been proposed as a complementary extension, emphasizing the importance of data and knowledge structures to be Cognitively interoperable, semantically Linked, contextually Explorable, intuitively Accessible, and human-Readable and -interpretable^5^. Together, FAIR and CLEAR highlight that future data and knowledge infrastructures must be usable not only by machines, but also by humans, including information architects, data analysts, and data and knowledge consumers.

Knowledge graphs, semantic graph schemata, and ontologies play a central role in this vision. They provide the technical, formal, and conceptual frameworks essential for organizing, integrating, and representing heterogeneous data and knowledge sources and promise a paradigm shift in how we access, navigate, comprehend, and derive insights from data and knowledge. Accordingly, they have become cornerstones across a wide range of domains and contexts, including semantic search, deep reasoning, knowledge discovery, decision support, natural language disambiguation, and data and knowledge integration^6–8^. In principle, FAIR and CLEAR knowledge graphs that are based on formal semantics promise a shift from isolated datasets toward interconnected, semantically enriched knowledge spaces that support both automated processing and human sense-making.

A critical step in constructing FAIR and CLEAR knowledge graphs is the transformation of information from diverse source models, such as relational databases, Comma-Separated Values (CSV) files, or natural language expressions, into structured graph-based representations. In practice, this often involves *semantic parsing* into the *Subject*―*Predicate*―*Object* triple syntax model of the Resource Description Framework (RDF), guided by ontologies and formal knowledge representation languages such as the Web Ontology Language (OWL), which is grounded in Description Logics. While such target models are well suited for automated reasoning, information integration, and information discovery through semantic search, and although both source and target model are models of the same referent system, they differ substantially in both structure and semantics^9^. As a result, creating a target model that preserves the source model’s meaning while enhancing its FAIRness, CLEARness, and overall machine-actionability remains a nontrivial challenge.

The semantic parsing challenge, and semantic modelling challenges in general, are not merely technical. At the core, they concern fundamental questions of *meaning*, *assertion*, *identity*, and *epistemic status* in data and knowledge representations. Scientific knowledge is often provisional, contextual, and contested. Statements may reflect hypotheses, interpretations, observations, or claims made by specific agents, rather than universally accepted facts. Yet many existing knowledge graph formalisms implicitly treat represented statements as asserted truths, making it difficult to model non-asserted content, epistemic attribution, disagreement, or evolving interpretations without introducing significant complexity or ambiguity.

In recent years, Large Language Models (LLMs) have attracted considerable attention for their ability to access, summarize, and generate scientific knowledge^10–16^. They have also been explored as tools for supporting the construction of knowledge graphs and ontologies, and they have been integrated with knowledge graphs to improve their own commonsense reasoning capabilities^17–24^. Despite this progress, the generation of high-quality, semantically precise ontologies and knowledge graphs suitable for scientific use still requires deep domain expertise and careful modelling decisions. Current LLM-based approaches cannot reliably replace ontology engineers or resolve fundamental representational challenges related to meaning, context, and epistemic status^25,26^.

Further complications arise from the lack of a unified semantic framework and standardized formalism for representing data and knowledge across knowledge graph technologies. Although a common semantic infrastructure is desirable, its realization is hindered by diverse standards, conflicting ontologies, differing purposes of the underlying models, and the inherent subjectivity of semantic interpretation. These challenges make truly seamless interoperability between knowledge graphs difficult, if not impossible, to achieve^9^. Moreover, the term “knowledge graph” itself encompasses a wide range of implications^7^, including labeled property graphs (e.g., Neo4J) or triple stores based on RDF. Additionally, not all RDF-based knowledge graphs are grounded in logic-based formalisms like OWL is in Description Logics. Although other logics such as First-Order Logic exist, they remain underused due to scalability and tooling issues.

Whereas OWL offers a robust and widely adopted framework, supporting formal semantics and reasoning as a W3C standard, it is often perceived as overly complex and difficult to use by non-experts, and as offering limited cognitive interoperability, thus lacking required CLEARness^5^. Conversely, labeled property graph approaches emphasize usability and flexibility but lack a shared formal semantic foundation, limiting their suitability for rigorous scientific applications, thus lacking FAIRness^5,9^. As a result, practitioners face persistent trade-offs between formal expressivity and human interpretability.

Balancing FAIRness with CLEARness remains a persistent challenge in the design of knowledge graphs. Formal knowledge representation languages such as OWL provide well-defined semantics and reasoning capabilities, but impose structural and epistemic constraints that can limit the representation of contextualized, uncertain, or non-asserted knowledge. Other graph-based approaches emphasize usability and intuitive modelling, but often lack shared semantics and principled interoperability. These tensions are symptomatic of a more general problem: existing knowledge graph technologies offer powerful mechanisms for representing facts, but provide limited conceptual support for representing *meaningful units of knowledge*, their epistemic status, and their identity as coherent, human-interpretable entities.

This paper introduces the *Semantic Units Framework* as a *technology-agnostic conceptual framework* for structuring data and knowledge in ways that support both FAIR and CLEAR Principles. Semantic units are conceived as semantically coherent, independently addressable units of meaning that explicitly capture what is *being said*, *how it is meant*, and *under what epistemic assumptions*. Rather than proposing a new logic, ontology language, or technical standard, the framework provides a conceptual abstraction that can be implemented across different database technologies, including RDF/OWL-based graphs, labeled property graphs, and tabular and relational database infrastructures.

OWL and Description Logics are used throughout the paper as a *paradigmatic formal setting*. This choice allows us to analyze representational limitations with precision and clarity, but the challenges discussed are not specific to OWL alone. Instead, they reflect broader issues that arise whenever databases are used to model meaning, assertion, and identity in complex data and knowledge infrastructures. The Semantic Units Framework is therefore not an extension of OWL expressivity, but a technology-agnostic conceptual framework through which existing formalisms can be combined, constrained, or selectively relaxed to better support human-oriented data and knowledge representation.

Importantly, this paper is *conceptual in nature*. It does not present a software prototype, a reference implementation, or a new reasoning formalism. Nor does it aim to replace existing standards or tools. Its contribution lies in articulating a coherent conceptual framework that integrates and systematizes existing ideas, such as FAIR and CLEAR Principles, FAIR Digital Objects, Named Graphs, nanopublications, and epistemic modelling, into a unified perspective on how data and knowledge can be structured for both machine-actionability and human understanding.

The remainder of the paper is structured as follows. Section ‘*Conceptual challenges in FAIR and CLEAR data and knowledge infrastructures*’ identifies and synthesizes key conceptual challenges that arise in FAIR and CLEAR data and knowledge infrastructures. Section ‘*OWL Expressivity as a paradigmatic challenge*’ focuses on limitations in current OWL-based representations as illustrative examples, while Section ‘*From OWL limitations to general challenges of representing meaning, epistemics, and identity*’ generalizes to more fundamental challenges related to meaning, assertion, epistemic attribution, fragmentation, and identity. Within the second chapter, the first section introduces the Semantic Units Framework and its core concepts and introduces instance-quantified resource categories, complementing OWL’s standard categories (*named-individual*, *class*, *property*) to enable richer representations. The second section revisits the identified challenges and demonstrates how they can be addressed by combining instance-quantified resources with semantic units, first in the context of OWL-specific limitations (Section ‘*Addressing paradigmatic OWL expressivity limitations*’) and then for the broader set of fundamental challenges (Section ‘*Addressing general challenges of meaning, epistemics, and identity*’). The Section ‘*Relation to FAIR Digital Objects and existing work*’ relates the framework to the concept of FAIR Digital Objects, nanopublications, and other existing work. The third chapter discusses scope, generality, and limitations of the framework, as well as the implications for future knowledge infrastructures, and concludes the paper with concluding remarks.

Claims regarding improved cognitive interoperability, as captured by the CLEAR Principle, are grounded in the structural properties of the proposed representation rather than in empirical usability testing. Semantic units explicitly delimit coherent units of meaning, expose propositional structure, and provide stable identifiers for statements and their aggregations. These properties directly address well-documented sources of cognitive load in triple-based representations, including semantic fragmentation, implicit context, and reliance on complex graph traversal patterns. As such, they provide a conceptual foundation for the framework described in the second chapter. While empirical user studies constitute an important direction for future work, the present contribution focuses on establishing the conceptual and representational foundations required for such evaluations.

### Notation and terminology

To ensure clarity and consistency, we briefly outline the notational conventions and terminology used throughout this paper.

We use the term *resource* to be something that is uniquely designated (e.g., a Uniform Resource Identifier: URI) and about which someone wants to say something. It thus stands for something and represents something someone wants to talk about. In RDF, the *Subject* and the *Predicate* in a triple are always resources, whereas the *Object* can be either a resource or a literal. Resources can be either properties, instances, or classes, with properties taking the *Predicate* position in a triple and with instances referring to individuals (=particulars) and classes to universals or kinds.

Throughout the text and figures, resources are displayed using human-readable labels rather than full URIs, with the implicit assumption that each class, instance, and property is associated with a unique identifier. When referring to ontology classes, we use regular underlined text, *italicsUnderlined* when referring to properties, and use ID numbers to specify each. ID numbers are composed of the ontology prefix followed by a colon and a number, e.g., *hasQuality* (RO:0000086). If the term is not yet covered in any ontology, we indicate it with an asterisk (*). Newly introduced classes and properties relating to semantic units have the ontology prefix SEMUNIT as in the class *SEMUNIT:has semantic unit subject*. They will be part of a future Semantic Unit ontology. We use ‘regular underlined’ to indicate instances of classes, with the label typically referring to the class label and the ID number to the class.

In the paper, we distinguish carefully between *statements* in natural language and *triples* or *triple statements* in RDF. The term *triple* is used exclusively to refer to *Subject–Predicate–Object* expressions in a knowledge graph, whereas *statement* refers to a linguistic or conceptual assertion that may or may not be asserted as true within the graph.

Finally, when discussing knowledge graph technologies, we refer to RDF/OWL-based graphs and labeled property graphs as representative and widely adopted paradigms. Other formalisms and logics exist, including n-ary representations and First-Order Logic-based systems, but are not considered in detail due to limited standardization and tooling support.

### Conceptual challenges in FAIR and CLEAR data and knowledge infrastructures

This section examines a set of conceptual challenges that arise in the representation of data and knowledge within FAIR and CLEAR knowledge infrastructures. These challenges concern fundamental issues of meaning, assertion, epistemic status, fragmentation, and identity, which become particularly salient when knowledge is represented in graph-based formalisms intended to support both machine-actionability and human-interpretability.

To make these challenges concrete, we analyze them through the lens of RDF/OWL-based knowledge graphs, which serve here as a *paradigmatic formal setting*. We focus on OWL-based modelling, because OWL provides a well-defined logical foundation and is widely adopted in scientific knowledge representation, making it a useful framework for exposing tensions between formal expressivity and the richness of human meaning. Moreover, beyond their established role in semantic search, reasoning, and integration, structured knowledge graphs are increasingly discussed as grounding mechanisms for LLMs, where external symbolic representations are used to constrain generation and reduce hallucination^26,27^. While most current approaches employ lightweight or heterogeneous graph representations, OWL-based knowledge graphs represent the most explicit and formally defined end of this spectrum, making them a particularly instructive setting for analyzing how meaning, assertion, and identity are made machine-actionable and human-interpretable. However, the limitations discussed in this section are not represented as shortcomings of OWL per se, but as illustrative pressure points that reveal more general representational challenges.

The first subsection focuses on challenges that arise when attempting to represent diverse categories of statements, complex relationships, and contextual information within OWL-based knowledge graphs. These challenges highlight recurring trade-offs between logical rigor, expressive adequacy, and cognitive interoperability.

The second subsection abstracts beyond the OWL-specific context to articulate a set of technology-independent challenges, including the representation of non-asserted content, epistemic attribution and disagreement, semantic fragmentation, and the identity of entities with rich internal granular structure.

This section is intentionally descriptive and analytical in nature. Its purpose is to map the conceptual problem space rather than to propose solutions. The Semantic Units Framework is introduced in the first section of the subsequent chapter as a technology-agnostic conceptual response to these challenges, with the second section returning to the issues identified here to demonstrate how they can be addressed in a systematic and principled way.

#### OWL Expressivity as a paradigmatic challenge

The following subsections describe challenges related to OWL-based knowledge graphs.

##### Foundational challenge: Unified representation of diverse categories of statements

Scientific communication relies on the ability to distinguish between different categories of statements, each expressing a distinct relationship between meaning, truth, generality, and reference. Knowledge graphs intended to support FAIR and CLEAR knowledge infrastructures must therefore be capable of representing not only entities and relations, but also the semantic nature of the statements in which these entities participate. This challenge is *foundational*, as it underlies many of the representational and epistemic difficulties discussed in the subsequent sections. At a minimum, scientific discourse routinely employs five fundamental categories of statements:

*Assertional statements* express concrete claims about individual entities and are intended to be either *true* or *false*^28,29^. They refer to actualities in the world, reflecting *what is the case*, and typically report observations, measurements, or recorded events. For example: *“By 27 February 2020, the outbreak of coronavirus disease 2019 (COVID-19) caused 82623 confirmed cases and 2858 deaths globally”* (p.568^30^). In knowledge graphs, such statements are commonly represented as relations between known instances (i.e., OWL:namedIndividual).

*Existential statements* express *what can be the case*, rather than what is the case. They assert that at least one instance of a given class satisfies a certain condition, without committing to universality^28,29,31^. For example: *“… SARS-CoV-*2 *… can cause acute respiratory distress syndromes (ARDS)”* (p.569^30^). Such statements are prevalent in empirical sciences like biology, where universal claims are often untenable because one can usually always find exceptions to the rule. Notably, existential statements are frequently inferred from assertional statements: observing a single black swan suffices to conclude that swans can be black.

*Prototypical statements* constitute a special subclass of existential statements. They describe *what is typically but not necessarily the case* for the instances of a class, capturing statistical regularities or default expectations rather than strict logical necessities. For example: “*In the presence of native predators, morphological defences typically consist of developing deeper, longer, and more pigmented tails,…*” (p. 8^32^). Prototypical statements are especially common in biology, ecology, and medicine, where exceptions are the norm rather than an anomaly (see also *cluster classes* and *fuzzy sets* in^33^).

*Universal statements* assert *what is necessarily or impossibly the case* for all instances of a class. They express established domain knowledge, such as scientific laws, hypothesis, theoretical commitments, and definitions (see also *essentialistic classes* in^33^). For instance, the infamous example of a universal statement that was considered true by scholars in Europe until the 17th Century^34^: “…*all swans are white*” (p. 4^35^); until in 1667, when the Dutch explorer Willem de Vlamingh discovered the black swan, *Cygnus atratus*, while exploring the coastlines of Western Australia.

*Lexical statements* concern linguistic entities themselves, discussing terms and their attributes as textual representational artefacts (cf. *terminological statement* sensu^28^; see also Ingvar Johannson’s distinction between *use* and *mention* of linguistic entities^36^). Lexical statements include labels, synonyms, human-readable definitions, and provenance metadata, in ontologies often represented using annotation properties.

While these categories are routinely distinguished in natural language and scientific reasoning, their faithful representation in knowledge graphs is far from straightforward. Consider a single conceptual example, “*white swan*”, expressed across different statement categories (ignoring lexical statements for now):

1. **Assertional**: “*Swan Anton is white*”, or “*This swan has quality this white*” (*this-to-this* relation, with ‘this’ being both a spatial and a temporal indicator, unambiguously designating an individual entity).
2. **Existential**: “*Swans can be white*”, or “*Some swan has quality some white*” (*some-to-some* relations, with ‘some’ in the sense of ‘there exists a’, which refers to one or more anonymous entities).
3. **Prototypical**: “*Swans are typically white*”, or “*Most swans have quality some white*” (*most-to-some* relations).
4. **Universal**: “*All swans are white*”, or “*Every swan has quality some white*” (*all-to-some* relations).

Although all four statements reuse the same conceptual vocabulary, they differ fundamentally in their semantic commitments. Accurately capturing these differences requires representational means that go beyond merely linking resources such as ‘*swan*’, ‘*white*’, and ‘*has quality*’.

**Fig. 1:**
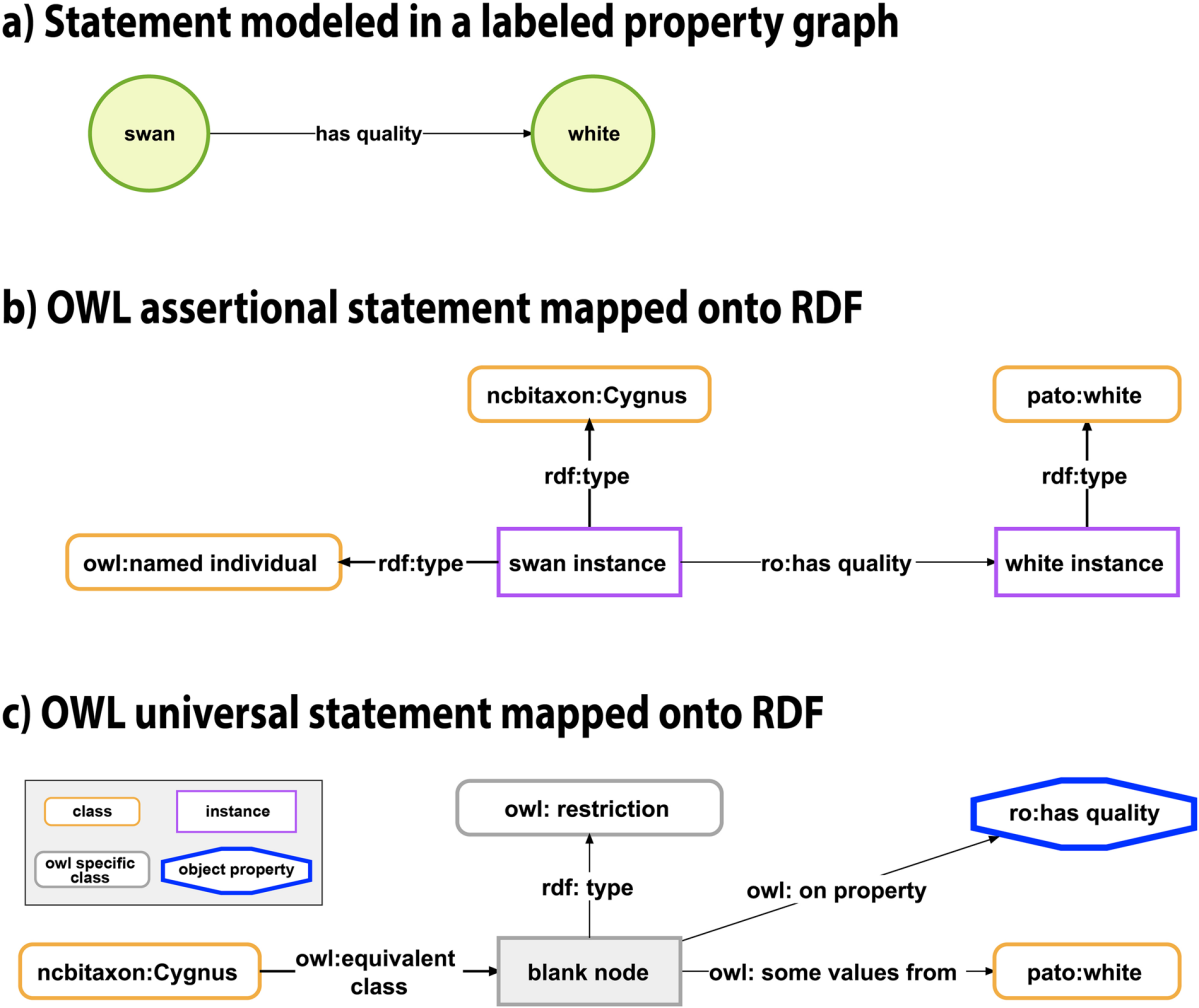
Comparison of statement categories as they can be documented in a labeled property graph and an OWL-based RDF graph. (**a**) A labeled property graph cannot formally distinguish assertional, existential, prototypical, and universal statements and leaves it to the user to interpret the displayed relation. In OWL, mapped onto RDF, two different types of statements can be distinguished: (**b**) The assertional statement *This swan has quality this white* and (**C**) the universal statement *All swans have quality some white*. However, the latter is typically not documented in a knowledge graph but is only expressed as a class axiom within an ontology.

Current graph-based formalisms struggle to accommodate this diversity in a principled way. Labeled property graphs offer intuitive and cognitively accessible representations, but they lack standardized formal semantics, leaving the interpretation of statement categories largely implicit and context-dependent. OWL-based knowledge graphs, grounded in truth-conditional Description Logics, provide formal semantics for certain statement categories, i.e., universal and assertional statements, but do so by embedding them into distinct logical strata, locating universal statements into the terminology box (TBox; see Fig. 1c) and separating them from assertional statements, which are located in the *domain of discourse* of the knowledge graph, i.e., the assertion box (ABox; see Fig. 1b)^37^. This design choice enables automated reasoning but limits the ability to explicitly represent other statement categories, such as existential and prototypical claims, that are central to scientific discourse.

Whereas representing the universal statement “*All swans are white*” in OWL results in a complex but semantically precise representation of the statement (Fig. 1c), a domain expert will likely have problems understanding it and would prefer the simpler and still intuitively comprehensible representation provided by a labeled property graph (Fig. 1a). In other words, the OWL expression lacks cognitive interoperability and thus CLEARness. Moreover, the OWL-based representation involves blank nodes, which makes basic SPARQL querying difficult^37^ and has the potential to cause other issues^38^ (see also Section '*Challenge: Representing complex class axioms*'). While solutions exist for representing universal statements in labeled property graphs, such as annotating edges with logical properties (e.g., existential restriction axioms), resulting in an object-property mapping^37,39^, there is no standardized W3C recommendation for mapping OWL into property graphs.

In summary, the diversity of scientific statements underscores the limitations of current graph modelling formalisms. Labeled property graphs comply with the CLEAR Principle but lack formal semantics, whereas RDF/OWL-based graphs provide for some statement types formal semantics but fall short in representing all statement types and fail in cognitive interoperability (for an overview of the differences between RDF/OWL-based knowledge graphs and labeled property graphs, see Table 1).

**Table 1:** Comparison of the advantages and shortcomings of knowledge graphs based on RDF/OWL and on labeled property graphs Comparison of the advantages and shortcomings of knowledge graphs based on RDF/OWL and on labeled property graphs^37,39^.

	RDF/OWL	labeled property graph
query language	SPARQL, W3C recommendation	various, none has a W3C recommendation
formal semantics and reasoning	OWL; however: 1. when mapped to RDF, OWL is usually very verbose, leading to unnecessarily complex graphs that are not intuitively comprehensible for a human reader and inhibit effective computation; 2. OWL is not the only mode of inference, and other frameworks besides Description Logics exist for inferencing	no unified set of standards (no W3C recommendation)―a standardized mapping of OWL to a property graph is still lacking; the data model of Neo4j is not based on a formal set theory or First-Order Logic
relating information directly to edges	only indirectly through reification, RDF-star, or Named Graphs	information can be directly related to relations, i.e., edges
distinction of assertional, existential, prototypical, and universal statements	formalized semantic distinction between assertional and universal statements; existential and prototypical statements are not modelled in OWL	no formalized semantic distinction

Importantly, these limitations should not be understood as idiosyncrasies or shortcomings of OWL alone. Rather, they reflect more general structural consequences of truth-conditional, logic-centric knowledge representation frameworks, in which statements are primarily treated as vehicles for inference rather than as first-class semantic objects with diverse epistemic roles. As a result, knowledge graphs often conflate *meaning* with *assertability* and struggle to preserve distinctions that are essential for human interpretation, exploration, and critical assessment.

This foundational challenge motivates the need for a more general representational perspective, one that can accommodate the full spectrum of scientific statements without presupposing that all of them must be asserted, universally valid, or logically tractable. The following sections examine additional challenges that arise from this tension and further illustrate why a shift in representational framing is required.

##### Challenge: Representing universal statements

Universal statements are central to scientific knowledge, expressing general laws, definitions, and constraints. They are frequently provisional, contested, or revised over time and therefore often function as objects of interpretation rather than as immutable facts. In knowledge graphs intended to support FAIR and CLEAR Principles, such statements must be referable, annotatable, and contextualizable.

In OWL-based knowledge graphs, universal statements are typically encoded as class axioms in the TBox. While classes are identified by URIs, the axioms defining them generally lack identifiers and cannot be referenced as independent graph elements. As a result, the formal semantic content of universal statements, despite being central to a class’s meaning, is not part of the explicit domain of discourse in a knowledge graph. Statements such as “*Author A challenges definition X*” or “*Assumption Y was revised in light of new evidence*” cannot be directly represented when the universal claims *X* and *Y* exist only implicitly within a class definition.

This limitation hinders the representation of provenance, epistemic attribution, and scientific disagreement regarding universal statements. Although annotation properties can be attached to classes, they do not allow specific axioms to be treated as first-class knowledge claims that can be compared, qualified, or debated.

And also, this challenge is not unique to OWL. It reflects a more general characteristic of truth-conditional representation formalisms, in which universal statements are encoded as background semantics rather than as explicit objects of discourse. This creates a structural gap between how universal knowledge functions in scientific reasoning and how it is represented in current graph-based systems.

##### Challenge: Representing complex class axioms

A persistent challenge in formal knowledge representation arises when class axioms require multiple components of a statement to refer to the *same* underlying entity. Such situations occur when expressing relationships that involve internal dependencies, so-called *triangular relationships*, where two or more relational paths must converge on a shared referent.

In OWL-based knowledge graphs, this challenge becomes apparent due to structural characteristics of Description Logics, including the tree model property of Description Logics and the use of blank nodes in RDF serializations. Universal statements defined in the terminology component (TBox) are often serialized in RDF using blank nodes to represent anonymous individuals (OWL:AnonymousIndividual), as shown in Fig. 1c. While blank nodes allow the construction of complex class expressions, they lack global identity and cannot be reliably referenced outside or even within the same axiom in a meaningful way.

As a result, it becomes difficult or even impossible to express co-reference within a single axiom when multiple relational components are intended to involve the same entity. Dependencies between different components of an axiom remain implicit, and formal semantics may interpret them as involving distinct anonymous individuals rather than a shared one.

This limitation has implications for representing empirical knowledge, as blank nodes undermine the findability, accessibility, explorability, comparability, expandability, and reusability of any expression modelled as TBox, which is why empirical data should be preferably modelled as ABox expressions (for a detailed discussion see^40^).

A concrete example is the class *antenna type 1*, which is defined as “*An antenna that is longer than the eyes of the same organism that possesses it*”, requiring that the antenna and the eye be explicitly linked to the same organism. In OWL, such an axiom can be expressed syntactically, but its formal interpretation fails to enforce this shared reference due to the reasons discussed. This becomes apparent when examining the Manchester Syntax^41^ of the corresponding axiom, where the phrase “*some multicellular organism*”, which represents an anonymous individual, appears twice:

‘has part some ((antenna and part of *some multicellular organism*) and has quality some (length and increased in magnitude relative to some (length and inheres in some (eye and part of *some multicellular organism*))))’.

Under OWL semantics, each anonymous individual is interpreted as a distinct entity. Consequently, the axiom permits unintended interpretations in which the antenna of one organism is compared to the eye of another^42^. While this issue manifests clearly in OWL, it reflects a more general representational limitation of tree-structured, truth-conditional formalisms. Whenever complex constraints depend on explicit co-reference within structured statements, formalisms that lack mechanisms for stable internal identity struggle to preserve the intended semantics. This poses challenges for representing empirically grounded knowledge, where internal relational consistency and contextual coherence are often essential.

##### Challenge: Representing negations and cardinality restrictions

Scientific data and knowledge frequently involve negations, absence statements, and cardinality constraints. Researchers routinely need to express that a property does *not* hold for a particular entity, or that a relation holds for *exactly*, *at most*, or *at least* a given number of instances. Accurately representing such statements is essential for empirical interpretation, data validation, and scientific communication.

**Fig. 2:**
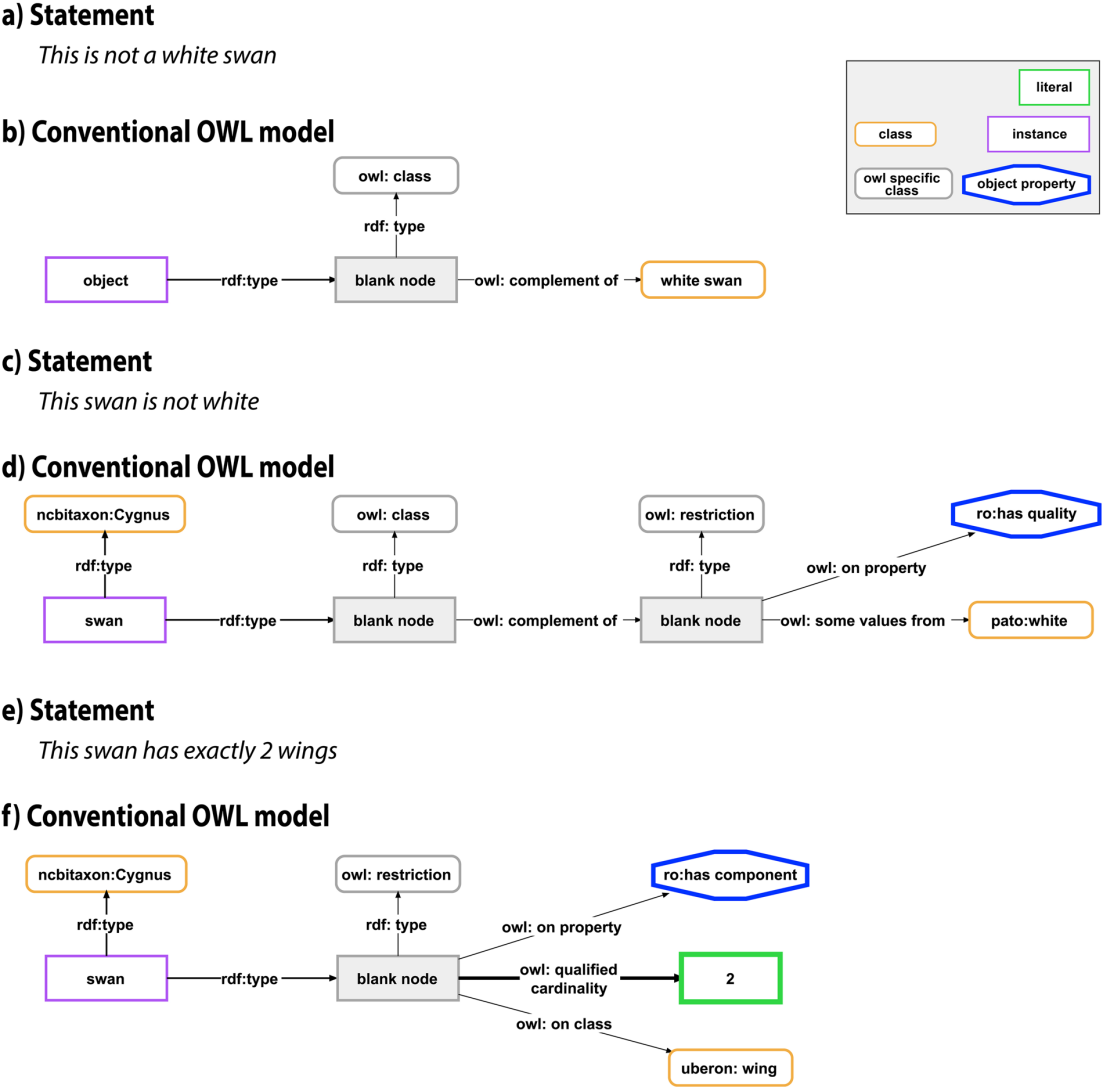
Modelling negations and cardinality restrictions for particular instances. (**a**) The assertional statement “*This is not a white swan*.” (**b**) The same statement translated into an OWL expression mapped to RDF. (**c**) The assertional statement “*This swan is not white*.” (**d**) The same statement translated into an OWL expression mapped to RDF. (**e**) The assertional statement “*This swan has exactly 2 wings*.” (**f**) The same statement translated into an OWL expression mapped to RDF. Note that all OWL models require at least one blank node.

In OWL-based knowledge graphs, negations and cardinality restrictions can be expressed formally, but doing so typically requires complex class-level axioms defined in the TBox. This is a direct consequence of OWL’s grounding in Description Logics and its adherence to the Open World Assumption (OWA), according to which the absence of a statement cannot be interpreted as its negation. As a result, instance-level negation, such as “*This swan is not white*”, cannot be represented as simple assertional statements, but must instead be encoded via class complements or related constructs, resulting in more complex and less CLEAR representations (cf. Figure 2).

A comparable situation arises for cardinality constraints. Statements such as “*This swan has exactly two wings*” cannot be expressed directly at the instance level, but must be translated into a class axiom involving a qualified cardinality restriction (Fig. 2f). While these representations are logically precise, they rely on complex syntactic structures, often involving blank nodes, that are difficult to interpret, author, or query, particularly for users without expertise in formal logic.

By contrast, labeled property graph models allow negations and cardinality constraints to be expressed in a more direct and intuitive manner, often as explicit edge annotations or constraints attached to individual nodes. However, these representations typically lack standardized formal semantics and therefore do not support rigorous logical interpretation or inference.

This tension highlights a broader representational challenge that extends beyond OWL. Truth-conditional, logic-based formalisms tend to encode negation and cardinality as global constraints rather than as first-class, contextual statements, while more user-friendly graph models sacrifice formal rigor for usability. As a result, existing knowledge graph technologies struggle to provide representations that are simultaneously expressive, cognitively interoperable, and semantically well-founded when it comes to negations and cardinality restrictions.

##### Challenge: The complexity of querying FAIR knowledge graphs

Interacting with a knowledge graph—whether to retrieve, add, update, or delete content—typically requires proficiency in specialized graph query languages, such as SPARQL for RDF/OWL-based graphs or Cypher for labeled property graphs like Neo4j. While these languages are expressive and powerful, they present a substantial barrier to entry. Moreover, effective querying requires not only command of query syntax but also a detailed knowledge of the underlying graph structure (schemata), ontologies, and modelling conventions. Even technically skilled users often find query construction time-consuming and error-prone, limiting the accessibility and broader adaption of knowledge graphs^43^.

This challenge directly affects findability and discoverability, two core requirements of FAIR and CLEAR knowledge infrastructures. When users cannot easily formulate queries that reflect their informational intent, relevant data may remain effectively inaccessible despite being formally well-structured. The problem exacerbated in large or heterogeneous knowledge graphs, where schema complexity and modelling variation further increase cognitive load.

Recent advancements in LLMs have demonstrated the potential to translate natural language questions into formal graph queries^10^. While promising, empirical studies indicate that current approaches perform reliably only for relatively simple queries. As query complexity increases, particularly with nested patterns, constraints, or contextual dependencies, performance degrades, and ambiguities in natural language frequently lead to incorrect or unintended results^11,12^. Moreover, LLM-generated queries often lack transparency and predictability, complicating validation and trust.

##### Challenge: Representing interrogative statements

Interrogative statements (i.e., questions) are fundamental to how humans interact with data and knowledge. They express information needs, guide exploration, and frame the interpretation of available evidence. In the context of knowledge graphs, interrogative statements are typically translated into formal queries, enabling machine-actionable retrieval and analysis. However, the questions themselves are not represented as part of the knowledge graph.

Current knowledge graph formalisms, including RDF/OWL-based graphs and labeled property graph models, lack native mechanisms for representing interrogative statements within the graph structure. Query languages such as SPARQL or Cypher operate externally to the graph, treating questions as procedural instructions rather than as semantic objects in their own right. As a result, interrogative statements remain outside the formal domain of discourse of the knowledge graph.

This limitation has several implications. Different types of questions, such as existential (“*Is there any…?*”), universal (“*Do all…?*”), comparative (“*Which is larger…?*”), or exploratory (“*What is known about…?*”), cannot be explicitly distinguished, categorized, or related to one another within the graph. Questions cannot be annotated with provenance, or evolving states of knowledge. Consequently, the role of questions as drivers of inquiry and sense-making is obscured in graph-based representations, and research questions, which play a substantial role in scientific communication, cannot be represented as questions in the graph.

##### Challenge: Representing standardized, topic-specific views as coherent knowledge objects

In many scientific, industrial, and regulatory domains, the exchange of data and knowledge is governed by standardized information formats that specify mandatory and optional content elements. Examples include material data sheets in manufacturing and automotive industries, such as those defined by the International Material Data System (IMDS), which prescribe required statements for the representation of a material’s composition, physical properties, and regulatory status. These standardized views function as authorative, reusable representations that are embedded in compliance workflows, exchange between organizations, and interpreted as coherent informational artefacts.

As knowledge graphs increasingly serve as backbone infrastructure for FAIR and CLEAR data and knowledge exchange, a corresponding requirement emerges: the ability to represent such standardized, topic-specific views within a broader graph structure. Conceptually, these views correspond to semantically coherent collections of statements that jointly describe a particular entity, process, or topic in a well-defined way. However, current knowledge graph formalisms lack mechanisms for representing these collections as first-class objects within the graph itself.

In RDF/OWL-based knowledge graphs, statements are represented as individual triples, and any higher-level coherence among them is implicit rather than formally modelled. While it is technically possible to *extract* subgraphs via queries, these subgraphs are not themselves entities in the graph’s domain of discourse. As a result, they cannot be directly referenced, annotated, or discussed within the same representational framework. Provenance, trust assessment, validity periods, or evaluative judgements can be attached to individual triples or Named Graphs, but not to semantically unified views understood as informational wholes. Comparable limitations arise in other graph-based formalisms, indicating that the difficulty of representing standardized views as first-class knowledge objects is not specific to OWL but reflects a more general representational issue.

The absence of first-class representations of standardized, topic-specific views has practical and conceptual consequences. It hinders reuse, comparability, and interoperability, as equivalent informational views cannot be reliably identified or exchanged across systems. It also limits cognitive interoperability, as users must reconstruct the intended scope and structure of a view from dispersed statements rather than interacting with it as a meaningful unit.

##### Challenge: Representing spatio-temporal and sequential information

In many scientific, historical, and real-world domains, the meaning, validity, and relevance of statements are intrinsically dependent on temporal, spatial, and sequential contexts. Assertions are often true only within specific time intervals, at particular locations, or relative to an ordered series of events. For example, the statement that *John F. Kennedy was the 35*^*th*^ *President of the United States* is meaningful only within a defined historical period (1961–1963) and derives part of its meaning from its position within a temporal sequence of presidencies. Similarly, a claim such as “*The G7 Summit took place in Fasano, Italy*” presupposes a specific date, location, and iteration within the recurring event series of G7 Summits.

Such contextual dependence is central to domains that deal with events, processes, and episodic phenomena, including scientific workflows and experimental protocols (e.g., multi-phase lab procedures), clinical interventions and treatment sequences, historical narratives, and environmental observations that are tied to specific times and places. In these settings, statements cannot be interpreted correctly without access to the temporal order, spatial anchoring, or process context in which they occur.

Representing this kind of spatio-temporal and sequential information within RDF/OWL-based knowledge graphs poses persistent challenges. OWL itself does not provide first-class constructs for time, space, or ordering. While ontologies for temporal and spatial concepts exist, their use typically requires the distribution of contextual qualifiers across multiple interconnected triples. As a result, what is conceptually a single, context-bound claim is fragmented into several triple-based assertions whose joint interpretation depends on external conventions or inference patterns.

RDF offers mechanism such as reification, singleton properties, RDF-star, or Named Graphs to associate metadata with statements, but these approaches introduce additional structural complexity and are frequently applied inconsistently across implementations. In practice, they often reduce cognitive interoperability, complicate querying, and obscure the boundaries of what constitutes a coherent spatio-temporally qualified statement. This becomes particularly problematic when temporal or spatial qualifiers apply not to a single triple, but to a group of interrelated triples describing a material entity, observation, or process.

More generally, this challenge reflects a structural tension inherent in triple-based, truth-conditional representation formalisms: contextual dimensions such as time, place, and sequence are not naturally treated as integral components of a statement, but must instead be attached indirectly through auxiliary structures. While OWL provides a clear illustration of this tension, the issue is not specific to OWL. Similar difficulties arise in other graph-based knowledge representation approaches whenever meaning depends on contextual cohesion across multiple triples and statements rather than on isolated facts, indicating that the underlying problem also relates to the problem of the fragmentation of meaning discussed in the Section ‘*Foundational challenge: Fragmentation of meaning and loss of coherence—the structural organization of meaning*’.

##### Challenge: Representing directive statements

Directive statements express prescriptive and procedural knowledge, i.e., *what ought to be done*, rather than descriptive knowledge about *what is the case*. They are central to procedural, normative, and goal-oriented domains, including medicine, scientific methods, industrial workflows, engineering maintenance, and everyday tasks.

Even seemingly simple processes can embed multiple directive components. For example, the task of *boiling an egg* involves a desired outcome (objective: a boiled egg), necessary resources and their specifications (e.g., 1 egg, 1 liter water, cooking pot), devices and their settings (e.g., stove and heat level), ordered action steps (e.g., “*heat water, then add egg*”), and sometimes conditional instructions (e.g., “*turn off the heat when the water boils*”). Directive statements may be explicit or implicit and may omit the agent performing the action (e.g., “*Boil the egg!*” implies an unspecified agent).

From a formal knowledge representation perspective, directive statements differ fundamentally from assertional statements: they do not describe states of the world, but prescribe intentions, goals, or plans for actions. Current knowledge representation formalisms, including RDF/OWL and labeled property graphs, are optimized for declarative (assertional) knowledge. They provide limited mechanisms for representing prescriptive content such as goals, plans, obligations, or conditional actions, and they cannot distinguish between directive variants that differ in scope, generality, or statistical likelihood.

Directive statements can span a spectrum of generality, paralleling assertional, existential, prototypical, and universal forms found in descriptive knowledge. For instance, one might prescribe that:

1. a specific entity must achieve a state: assertional directive (“*Make: Swan Anton is white!*”),
2. some entity must satisfy a condition: existential directive (“*Make: Some swan is white!*”),
3. most entities must meet a requirement: prototypical directive (“*Make: Most swans are white!*”), or
4. all entities must conform to a directive: universal directive (“*Make: Every swan is white!*”).

Despite the clear distinctions in intent and generalization, current knowledge graph formalisms provide no principled way to encode these modalities as part of the graph itself. This gap limits the capacity of knowledge infrastructures to support prescriptive reasoning, procedural planning, or normative analysis as well as the documentation of methods, recipes, algorithms, and other plan specifications.

##### Challenge: Representing directive conditional statements

Directive conditional statements combine a prescriptive action with a conditional premise, expressing what must be done when a particular condition holds. They occur frequently in scientific, procedural, and instructional contexts, and involve two core elements: an assertional statement in the condition (the *if-clause*) and a directive statement in the consequence (the *then-clause*).

For example: “*Add 2* *kg of potatoes to 10 liters of water if the water has a temperature of 90* *°C*”. The if-clause asserts a factual condition (“*the water has a temperature of 90* *°C*”), and the then-clause prescribes an action (“*add 2* *kg of potatoes to the water*”). Similar patterns appear across domains: in medicine (“*Administer 500* *mg of drug X if systolic blood pressure exceeds 160* *mmHg*”) or engineering (“*Open valve B if pressure in tank A exceeds threshold*”). Everyday procedural knowledge, recipes, laboratory protocols, and instructional content also rely heavily on such statements.

Directive conditional statements are a specialized subclass of conditional statements. Not all conditional statements are prescriptive; many are purely logical, analytical, or descriptive. For example, a philosophical proposition such as “*There is no X that is good AND not beautiful*” can be reformulated as conditional statement: “*If X is good, then X is beautiful*”^44^, which, in turn, may be expressed as the universal statement “*Everything that is good is also beautiful*”^44^. These transformations illustrate how conditional logic supports reasoning across both descriptive and prescriptive knowledge spaces.

Conditional statements can be compound, involving Boolean operators (AND, OR, XOR, NOT, EQUAL), further increasing their complexity. Accurately representing such structures requires capturing relationships not only between entities, but between statements themselves.

Current graph-based semantic technologies, including RDF/OWL-based graphs and labeled property graphs, provide limited support for representing higher-order relationships of this kind. Encoding a conditional relationship between two full statements, rather than simple entity-level relations, quickly becomes unwieldy using blank nodes, Named Graphs, or singleton properties. As a result, despite their prevalence and importance, directive conditional statements remain largely unrepresented in mainstream knowledge graph systems.

##### Challenge: Representing logical arguments

Logical arguments constitute a fundamental structure in scientific reasoning. They allow researchers to justify claims, generate hypotheses, and draw conclusions from evidence. Formally, an argument connects premises to a conclusion by way of a specific mode of reasoning—deductive, inductive, or abductive. Each argument type typically comprises three semantic components:

- *Rule-clause*: a general statement or principle.
- *Case-clause*: a particular fact or observation expressing an underlying classification of a given entity.
- *Result-clause*: a resulting observation of a particular property of the given entity.

Depending on the direction and modality of reasoning, the arrangement of these clauses shifts, resulting in three canonical patterns^45^:

- *Deductive argument*: A universal statement as the rule-premise (“*All swans are white*”) combined with an assertional statement as the case-premise (“*Anton is a swan*”) yields an assertional statement as the result-conclusion (“*Anton is white*”) that is *necessarily true*.
- *Inductive argument*: An assertional statement as the case-premise (“*Anton is a swan*”) combined with an assertional statement as the result-premise (“*Anton is white*”) yields a universal statement as the rule-conclusion (“*All swans are white*”) that is *probable* (*verisimilar*).
- *Abductive argument*: An assertional statement as the result-premise (“*Anton is white*”) combined with a universal statement as rule-premise (“*All swans are white*”) yields an assertional statement as case-conclusion (“*Anton is a swan*”) that is *possible* (*plausible hypothesis*).

All three argument types are structurally conditional, with premises forming the *if-clause* and the conclusion serving as the *then-clause*. The modality of the conclusion varies: *necessary* in deduction, *probable* in induction, and *possible* in abduction.

Despite their centrality in scientific discourse, logical arguments are poorly supported in current knowledge graph formalisms. RDF/OWL-based graphs and labeled property graph models provide no native constructs for multi-premise arguments, inference modality, or compound dependencies. Existing formalisms already struggle with representing universal and directive conditional statements (see '*Challenge: Representing universal statements*' and '*Challenge: Representing directive conditional statements*'). As a result, arguments are often encoded externally, through rule engines, informal notes, or application-level conventions, rather than as first-class knowledge objects within the graph. This limitation reduces the capacity of knowledge graphs to capture the inferential structure that underpins scientific reasoning and analytical workflows.

##### Synthesis of the OWL-related challenges

Taken together, the challenges discussed in this section reveal a recurring representational gap in contemporary knowledge graph formalisms. While OWL serves as a paradigmatic reference point due to its logical rigor and widespread adoption, the issues identified here are not confined to any specific technology or formalism. Rather, they reflect a more general tension: knowledge graphs are optimized for encoding isolated, truth-conditional statements, yet they lack native mechanisms for representing dependencies between statements, model distinctions, contextual constraints, and structured forms of reasoning. As a result, many forms of scientific, procedural, and argumentative knowledge remain difficult to represent in a way that is both machine-actionable (FAIR) and cognitively interoperable (CLEAR). These limitations motivate the need to abstract beyond individual formalisms and articulate the underlying conceptual challenges more explicitly in the following section.

#### From OWL limitations to general challenges of representing meaning, epistemics, and identity

The challenges identified in Section ‘*OWL Expressivity as a paradigmatic challenge*’ point beyond OWL-specific modelling constraints toward more fundamental representational tensions in knowledge graph design. Most graph-based formalisms treat meaning as flat and truth-conditional, prioritizing the representation of isolated statements implicitly assumed to be true. Human knowledge, by contrast, is deeply contextual and epistemic, involving claims that may be asserted or merely reported, beliefs held by specific agents, disagreements between sources, and entities whose identity is constituted by rich internal structure rather than by atomic facts.

While OWL makes these tensions particularly visible due to its formal rigor, the underlying representational gaps are method-independent. In the following subsections, we therefore abstract from OWL to articulate four general challenges concerning the representation of (i) non-asserted content, (ii) epistemic attribution and disagreement, (iii) semantic fragmentation, and (iv) the identity of entities with rich internal granular structure.

Together, these challenges can be understood as different facets of a single underlying problem: contemporary knowledge graphs lack explicit mechanisms for representing contextuality and semantic coherence as a first-class aspect of data and knowledge. This aligns with emerging discussions around *context graphs* and *holistic knowledge graphs*, which emphasize the need to represent scoped, perspective-dependent, and semantically coherent units of meaning rather than flat collections of triples. However, such discussions typically remain at the conceptual level and lack a unifying representation framework.

The combined analysis of the challenges in the previous section and this section delineates the conceptual problem space that motivates the Semantic Units Framework introduced in the first section of the '*Results*' Chapter.

##### Foundational challenge: Non-asserted content—the implicit truth-value of claims

A fundamental challenge in the representation of data and knowledge concerns the treatment of *non-asserted content*, i.e., statements that are mentioned, reported, hypothesized, believed, or attributed to an agent, without being endorsed as “true” by the knowledge graph itself. In scientific contexts, it is essential to distinguish between *the act of representing a claim* and the *assertion of a fact*.

However, most knowledge graph formalisms implicitly conflate *representation* with *assertion*. This reflects what Johansson^36^ identifies as a failure to distinguish between linguistic/social artefacts and the biological reality they aim to represent. In a standard RDF-based system, the presence of a triple is interpreted as an assertion that contributes to the model of the world described by the graph. Therefore, the system lacks a native mechanism to treat a statement *as an object of discourse* and thus something that can be talked about, attributed, or evaluated, without simultaneously committing to its truth. This makes it difficult to model statements such as “*Person A claims that X holds*” without the graph itself asserting both *A* and *X* as parts of the world model.

A variety of technical workarounds have been proposed to address this issue, including RDF reification^46^, Named Graphs^47^, RDF-star^48^, and provenance vocabularies. While these approaches enable statements to be referenced or annotated, they largely treat statements as technical artefacts rather than epistemic objects. As a result, they tend to be verbose, cognitively opaque, or insufficiently precise with respect to their epistemic status. In particular, they do not reliably prevent unintended truth propagation. Even when a statement is reified or placed in a separate graph, its propositional content often remains accessible to queries and reasoning processes as if it were an established fact. By failing to isolate non-asserted content, current infrastructures struggle to support the “*Expressing Without Asserting*” (EWA) requirements necessary for handling the uncertainty and interpretative nature of scientific discourse^49^.

This problem is not specific to RDF or OWL. It reflects a more general limitation of truth-conditional, statement-centric representation frameworks, which are optimized for encoding what is taken to be the case, rather than for representing claims, beliefs, or reports *as such*. Human knowledge, by contrast, is deeply contextual and attributional. Statements are made by agents, under specific conditions, with varying degrees of commitment, confidence, and contestability. The inability to represent non-asserted content as first-class elements therefore constitutes a major obstacle to capturing epistemic nuances, handling disagreements, and supporting interpretative or exploratory uses of knowledge graphs.

This challenge also underlies several downstream issues addressed in the following sections, including the representation of epistemic attribution and disagreement, the coexistence of contradictory claims, and the contextual interpretation of knowledge. Addressing non-asserted content thus emerges as a foundational requirement for knowledge infrastructures that aim to support both FAIR machine-actionability and CLEAR human-interpretability.

##### Challenge: Epistemic beliefs and disagreement—the epistemic stance of claims

Scientific knowledge production is inherently epistemic. Researchers do not merely record facts. They formulate hypotheses, report observations with varying degrees of confidence, disagree with competing interpretations, and revise their beliefs over time. Scholarly discourse therefore consists not only of statements about the world, but also of *epistemic stances* toward those statements, i.e., expressions of belief, doubt, rejection, agnosticism, or conditional acceptance, often attributed to specific agents or sources.

Representing such epistemic beliefs and their attribution is essential for capturing the dynamics of scientific knowledge. Typical scenarios include conflicting claims from different sources, evolving measurements under changing experimental conditions, or explicit disagreement between researchers regarding the validity of a hypothesis. In these cases, the coexistence of multiple, potentially incompatible claims is not an error, but a faithful reflection of the state of knowledge at a given time.

A fundamental difficulty arises because epistemic attribution presupposes the ability to represent statements *without asserting their truth*. To state that “*Agent A believes X*,” “*Agent B rejects X*,” or “*Agent C remains undecided about X*,” the content of *X* must be available as an object of discourse without being endorsed by the knowledge graph itself. If the mere representation of *X* already commits the graph to its truth, epistemic distinctions collapse and disagreement becomes logical inconsistency, plural perspectives cannot coexist without undermining reasoning. Current knowledge graph formalisms struggle with this requirement.

While attribution mechanisms exist for RDF/OWL-based knowledge graphs, such as reification, provenance annotations, or Named Graphs, they do not reliably suspend assertion or entailment. As a result, epistemic information is layered on top of statements whose truth is already implicitly assumed. Labeled property graphs offer more modelling flexibility, but similarly lack formal mechanisms for representing epistemic modalities such as belief, doubt, or disagreement in a principled and interoperable way. This structural limitation has led to the development of alternative models like nanopublications^50–53^ and micropublications^54^, which attempt to wrap claims in an administrative layer to protect the global graph from inconsistency. However, as noted in previous section, these remain primarily syntactic wrappers rather than a fundamental shift in how the graph interprets the propositional content of the claim itself.

While scholarly discourse has been characterized as a network of connected hypotheses and epistemic stances^55–57^, epistemic disagreement remains difficult to represent without either introducing logical inconsistency or resorting to ad hoc modelling conventions. Conflicting claims are often externalized into textual descriptions, separate databases, or narrative interpretations, rather than being integrated into the knowledge graph itself. This limitation hinders the representation of scientific controversy, uncertainty, and evolving consensus, and restricts the ability of knowledge graphs to function as faithful records of scholarly discourse rather than as static repositories of presumed truths.

At a more general level, this challenge reflects a mismatch between human knowledge practices, which are inherently perspectival, attributional, and provisional, and graph-based representation frameworks that are optimized for encoding decontextualized, truth-conditional statements. Addressing epistemic beliefs and disagreement therefore requires representational mechanisms that can distinguish between *what is said*, *who says it*, and *whether it is endorsed*, without collapsing these dimensions into a single layer of asserted facts.

##### Foundational challenge: Fragmentation of meaning and loss of contextual coherence—the structural organization of meaning

A recurring challenge in contemporary knowledge infrastructure designs is the fragmentation of meaning across large numbers of fine-grained statements. In many graph-based representation formalisms, knowledge is decomposed into minimal atomic meaning-carrying units, i.e., triples, without an explicit notion of higher-level semantic cohesion. While this atomization supports flexible recombination and formal reasoning, it often comes at the cost of loss of contextual integrity and human interpretability.

In RDF-based knowledge graphs, this fragmentation is a direct consequence of the triple as the fundamental representational unit. Complex material entities, processes, observations, or information entities are described by distributing their meaning across potentially hundreds of connected triples. The resulting graph structure provides no native mechanism to indicate which triples jointly constitute a coherent semantic whole, nor to distinguish core defining information from peripheral or contextual details. As a result, meaning emerges only implicitly through graph traversal, schema interpretation, or external conventions. This decontextualization is a known bottleneck in the usability of linked data, where the *atomic* nature of triples fails to provide the structural *rails* necessary for complex interpretations^58^.

This fragmentation poses several practical and conceptual problems. For human users, it becomes difficult to recognize which statements belong together, to reconstruct the intended scope of an entity or event, or to understand the context in which a claim was made, ideally supporting contextual exploration of the contents of the graph^5^. For machines, especially in downstream analytical or AI-driven applications, fragmented representations complicate the extraction of semantically coherent units of information for reasoning, explanation, or decision support. Query results often return collections of triples rather than structured, meaningful objects, placing a substantial interpretative burden on consuming systems.

Recent discussions in industry and applied AI contexts, often framed under the notion of *context graphs*, reflect growing dissatisfaction with this situation. These discussions emphasize the need to make contextual dimensions such as temporal validity and provenance explicit and structurally integrated^59^. While such proposals vary in terminology and scope, they converge on the observation that flat collections of triples are insufficient to capture the structured, situation-dependent nature of real-world knowledge.

This fragmentation problem is not limited to RDF or semantic web technologies. Similar issues arise in other graph-based and relational representations, whenever complex entities or situations are modelled as aggregations of independent statements without an explicit unifying structure. The challenge, therefore, is not merely technical but conceptual, how to preserve semantic wholeness and contextual coherence in representations that are fundamentally decompositional.

This loss of semantic cohesion becomes especially problematic in knowledge infrastructures intended to support FAIR and CLEAR principles. Fragmented representations impede findability, do not support contextual exploration, and hinder reuse by obscuring the boundaries of meaningful information units, and they undermine cognitive interoperability by forcing users to mentally reconstruct context from scattered elements. Addressing fragmentation thus emerges as a prerequisite for knowledge infrastructures that aim to function not only as repositories of statements, but as intelligible representations of granular and often highly complex systems of meaning.

##### Challenge: Identity ambiguity—the collapse of representational unity under high granularity

A fundamental challenge in contemporary knowledge graph design concerns the representation and identification of entities that exhibit rich internal structure, contextual depth, and compositional complexity. Examples include scientific experiments, industrial processes, material entities, datasets, observations, workflows, or other composite entities whose meaning is constituted not by a small set of attributes, but by an interconnected structure of relationships, constraints, parts, and contextual information.

In classical RDF and OWL practice, a conceptual distinction is typically drawn between an entity in the world (the *thing*) and a representation describing that entity (e.g., a graph fragment)^46,59^. While this distinction is theoretically useful, it becomes increasingly problematic in knowledge graphs where entities are not merely referenced, but extensively *constructed* through their digital descriptions. This tension is exemplified by the *Identity Crisis* of the Semantic Web, where it is often formally ambiguous whether a URI identifies a non-information resource (the *thing*) or an information resource (the *description*)^60^. In such cases, it is often unclear whether an identifier refers to the entity itself, to a document describing it, to a particular observation of it, or to a partial representation embedded in a larger graph.

This tension has long been recognized in Linked Data research as an *identity management problem*. In particular, the widespread misuse of identity predicates such as *OWL:sameAs* has highlighted the gap between formal identity semantics and practical modelling needs. Empirical analyses have shown that *OWL:sameAs* is frequently employed to signal similarity, relatedness, or referential overlap rather than strict identity, leading to unintended entailments and inconsistent interpretations across datasets^61^. These issues are not merely syntactic errors, but symptoms of deeper ambiguity about what exactly is being identified.

The problem is exacerbated by the highly fragmented nature of triple-based representations. As discussed in the previous section, complex entities are typically described through large numbers of fine-grained triples distributed across a graph, without an explicit boundary indicating which triples jointly constitute the entity as a coherent whole. When contextual information such as provenance, temporal validity, experimental conditions, or process structure is similarly fragmented, the boundary of the entity becomes increasingly unclear. As a result, multiple overlapping graph fragments may all plausibly be interpreted as referring to “the same” entity, even though they differ in scope, granularity, or perspective.

For entities with minimal structure, such ambiguity may remain manageable. However, for granular entities, such as processes, experiments, datasets, or composite material systems, identity becomes inseparable from questions of internal structure and contextual completeness. Determining whether two representations refer to the same entity, to different stages of the same entity, or to distinct but similar entities often depends on which relationships are considered constitutive and which contextual dimensions are deemed essential. In applied fields such as materials science, this has led to the development of ontology-driven frameworks that attempt to structure entire process chains as coherent semantic models rather than collections of isolated events^62^. In the absence of explicit semantic constructs to express such boundaries, identity decisions become modelling conventions rather than semantically grounded distinctions^63^.

Existing RDF-based mechanisms such as RDF reification^46^, Named Graphs^47^, and RDF-star^48^ offer ways to annotate statements or group triples, but they primarily address the problem of statement-level metadata rather than the identity of complex entities. While these mechanisms allow additional information to be attached to graph fragments, they do not provide principled guidance on how identifiers should be assigned to entities whose meaning is distributed across such fragments. In practice, this often leads to the proliferation of auxiliary identifiers, parallel representations, or ad hoc alignment strategies, increasing modelling complexity and the risk of epistemic ambiguity^7^. Even sophisticated algorithmic approaches to graph alignment, such as those based on *bisimulation*, struggle to resolve identity when the underlying graph fragments lack explicit semantic boundaries^64^.

These identity challenges become particularly pronounced in knowledge graphs intended to support FAIR and CLEAR principles. For data to be findable, reusable, and cognitively interoperable, entities must have stable, intelligible identities that align with how users conceptualize and reason about them. When identity is unclear or inconsistently applied, knowledge graphs risk becoming collections of loosely connected descriptions rather than reliable representations of structured knowledge.

Recent discussions on the evolution of knowledge graphs, often framed in terms of *holistic* or *context-aware* graph architectures, reflect growing awareness of these limitations^65^. This perspective aligns with long-standing efforts in semantic process modelling to annotate process artefacts with structured descriptions that persist across the entire modelling and execution lifecycle^66^. This shift mirrors the emergence of *Digital Twins* as complex proxy models in industrial contexts, where the digital construct must maintain a stable identity even as its constituent descriptions evolve. This further exposes the inadequacy of traditional representation assumptions that separate the *thing* from the *description* without providing mechanisms to reconcile identity with internal structure and context.

Taken together, these observations suggest that identity ambiguity in knowledge infrastructures is not an isolated modelling issue, but a structural consequence of fragmentation combined with increasing representational granularity. Addressing the identity of such complex entities therefore requires rethinking how coherence, context, and internal structure are treated within knowledge representations. This suggests a shift toward a *holonic* architecture, where information units are treated simultaneously as autonomous wholes and as integrated parts of larger systems^67,68^. Such a perspective aligns with recent industry discussions on the evolution of *composite* or *holistic* knowledge graphs, which move beyond flat triple-stores toward structures that can maintain internal integrity while remaining globally connected.

## Results

### The semantic units framework

The challenges identified in previous chapter reveal a recurring limitation of contemporary knowledge graph formalisms. While they are highly effective at encoding isolated, truth-conditional statements that are typically realized through binary relational structures, they lack explicit mechanisms for representing semantic coherence (including relations that are conceptually n-ary, i.e., relations between more than two entities), epistemic status, contextual scope, and semantically meaningful collections of triples as first-class elements of a graph. Consequently, many forms of scientific, procedural, and argumentative knowledge remain difficult to represent in ways that are both machine-actionable (FAIR) and cognitively interoperable (CLEAR).

To address this gap, we introduce the *Semantic Units Framework*, a technology-agnostic representational framework designed to support the modular, contextual, and epistemically explicit representation of knowledge in knowledge infrastructures. The framework is grounded in two interrelated concepts that together extend the expressive capabilities of existing knowledge graph paradigms without replacing them.

The first concept is that of *semantic units*, i.e., identifiable, bounded units of meaning that group together semantically coherent statements and can be referenced, annotated, and related to one another as first-class objects. Semantic units have been introduced in previous work^69^ and provide explicit boundaries for meaning, enabling the representation of assertions, claims, procedures, arguments, and other complex knowledge structures as modular and reusable components within a knowledge graph.

The second concept is a *novel representational convention* that introduces four instance-quantified categories of resources, i.e., *some-instance*, *most-instances*, *every-instance*, and *all-instances* resources. These categories extend the standard resource model of RDF and OWL-based systems beyond *named-individuals*, *classes*, and *properties*, enabling more faithful representations of scientific generalizations, universal statements, prototypical claims, and other forms of data and knowledge that are central to human reasoning but poorly supported by existing graph-formalisms.

Crucially, these two concepts are not independent. Semantic units provide the structural and epistemic boundaries within which instance-quantified resources acquire their intended meaning, while the instance-quantified resource categories supply the expressive primitives required to model the diverse statement types identified in the sections addressing the various challenges. Together, they enable the explicit representation of context, epistemic status, and semantic coherence as integral aspects of knowledge graph content.

The Semantic Units Framework is intentionally *technology-independent*. It does not prescribe a specific ontology language, query language, or storage technology. Instead, it defines a representational layer that can be realized across different graph-based or relational technologies, including RDF/OWL-based knowledge graphs, labeled property graphs, and relational or tabular database systems. OWL and RDF are used in subsequent sections as concrete exemplars because of their formal rigor and widespread adoption, not because the framework is limited to them.

This section introduces the core principles of the Semantic Units Framework and defines its central representational constructs. As such, it builds on, but also substantially extends, the semantic unit concept introduced in^69^. While the original work was focused on formalizing semantic units as modular carriers of meaning and demonstrating their applicability within graph-based infrastructures, the present work contributes (i) a systematic challenge-driven motivation grounded in FAIR and CLEAR requirements (Section '*Conceptual challenges in FAIR and CLEAR data and knowledge infrastructures*'), (ii) a technology-agnostic generalization beyond RDF/OWL and labeled property graphs, (iii) an innovative approach that combines the semantic unit concept with the here newly introduced instance-quantified resource categories, and (iv) an explicit taxonomy of semantic unit types designed to address the challenges. By integrating these two concepts—semantic units and new instance-quantified resource categories—we provide a practical approach to systematically addressing the sixteen conceptual challenges identified above. This integration supports the development of more semantically rich and cognitively interoperable knowledge graphs.

#### Core principles of the semantic units framework

The Semantic Units Framework is grounded in a set of design criteria that jointly define a representational stance toward data and knowledge in FAIR and CLEAR infrastructures. These criteria are conceptual commitments rather than syntactic prescriptions, and they address the recurring representational limitations identified above. At the heart of the framework lies the *Semantic Modularisation Principle*^70^, derived from the observation that meaningful complex systems, like biological systems and natural language, are structured hierarchically into semantically coherent, modular meaning-building-blocks.

The criteria below operationalize the Semantic Modularization Principle by making explicit the representational commitments of the framework. They collectively motivate why data and knowledge must be represented as semantically bounded units, rather than as unstructured collections of atomic triples in graph-based systems or atomic data points and rows in relational and tabular systems.

##### Semantic boundedness criterion: explicit boundaries of meaning

Meaningful knowledge is not a cloud of separate assertions but a set of statements that together form a coherent object of discourse. The Semantic Modularisation Principle holds that knowledge representations must reflect this coherence by organizing information into *explicit*, *bounded semantic units*, each belonging to a specific class of information. This makes the boundaries of meaning machine-addressable and human-intelligible, supporting both discovery and interpretation as well as contextual exploration. Without such boundaries, coherence must be reconstructed implicitly through ad hoc conventions, undermining findability, interpretability, and reuse.

##### First-class statementhood criterion: statements as referable objects

In scientific and analytical practice, statements (including n-ary statements that require several triples when represented in a graph) function not only as carriers of truth but as objects of epistemic discourse. Researchers publish claims, attribute statements to sources, and debate interpretations. To support this, the framework treats *statements as first-class entities* that can be referenced, attributed, compared, and related independently of their asserted truth. This enables knowledge infrastructures to represent claims, hypotheses, contradictions, and beliefs as structured elements of the infrastructure rather than buried in annotation layers or external metadata stores.

##### Epistemic neutrality criterion: decoupling representation from endorsement

A robust representational framework must distinguish between the existence of a statement in the knowledge base and its epistemic status (*asserted*, *unasserted*, *reported*, *tentative*). Without this separation, representing disagreement or contested claims can collapse into logical inconsistency. The Semantic Units Framework therefore commits to *epistemic neutrality*, where the presence of a statement in the infrastructure does not by itself confer endorsement. Instead, epistemic qualifiers and attributions are explicit and orthogonal to propositional content.

##### Context as structure criterion: integrate contextuality into representation

Contextual information, including provenance, temporal scope, locality, conditions of applicability, methodological parameters, and information granularity, is not auxiliary add-on metadata but is constitutive of meaning. The Semantic Units Framework therefore treats context as a *structural element*, not an afterthought. This principle ensures that the conditions under which a claim is made, the evidence supporting it, and its scope of relevance are integral to how semantic units are defined and interrelated.

##### Identity through semantic coherence criterion: holistic entities

Many entities of interest (e.g., experiments, processes, material entities) derive their identity not from isolated properties but from the structured granular relations among their constituent parts. The Semantic Units Framework treats *identity as emerging from semantic coherence based on the entity’s intrinsic granular structure*. An entity is thus identified by the organization of statements that describe it as a meaningful whole. This principle responds to the identity ambiguity caused by fragmentation, ensuring stable reference even for richly structured granular entities by anchoring their identity to semantically bound units.

Together, these criteria establish a representational layer that prioritizes semantic integrity, epistemic clarity, and contextual coherence. They frame *semantic units* not as a specific technology or syntax but as the logical architecture necessary to support the representation of rich, complex, and contextually grounded knowledge. In this way, the Semantic Units Framework equips knowledge infrastructures to faithfully represent a broader range of scientific and analytical forms of information than conventional infrastructures, including knowledge graphs, typically allow.

#### Semantic units as modular units of meaning

**Fig. 3:**
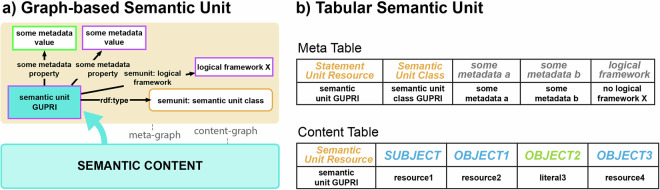
Core architecture of a semantic unit, exemplified within an OWL/RDF framework and a tabular/relational framework. (**a**) A graph-based semantic unit, with the unit’s semantic content (the meaning-carrying triples are not shown) being stored in a Named Graph, constituting the unit’s content-graph. The Named Graph’s GUPRI is at the same time also the GUPRI of the semantic unit (indicated by the blue arrow). All contextual information relating to the semantic unit itself, including the class it instantiates, its provenance, and other metadata, are stored in the unit’s meta-graph. (**b**) A semantically equivalent semantic unit, implemented using a tabular or relational database. The information from the content-graph is organized in the content table, and the information from the meta-graph in the meta table of the corresponding semantic unit class.

Compliant with the Semantic Modularisation Principle, semantic units are modular, *self-contained data and knowledge structures* that represent a coherent *unit of meaning* within a knowledge infrastructure (Fig. 3). This concept of a semantic unit is based on previous work^69^. Each semantic unit carries *semantic content* either directly, as in the case of a single statement (=statement unit), or indirectly, by referencing a defined set of other semantic units (=compound unit). Content within a semantic unit is *interpretable as a meaningful whole* by decoupling its representation in user interfaces (UIs) from its documentation in databases, supporting human comprehension and machine-actionable operations alike.

Each semantic unit is assigned a *Globally Unique Persistent and Resolvable Identifier (GUPRI)*, which identifies both the unit itself and the semantic content it carries. The GUPRI also instantiates a corresponding semantic unit class, thereby classifying this content (Fig. 3). By design, information relating to the unit’s content is *clearly separated from the contextual and organizational metadata* associated with the unit itself.

This distinction between content and context can be realized in multiple ways, depending on the underlying technology. In graph-based infrastructures, the semantic content can be encapsulated in a *Named Graph*, forming the *content-graph*, with the Named Graph’s GUPRI also providing the unit’s GUPRI (indicated by the blue arrow in Fig. 3a). The content-graph contains all triples modelling the semantic content, so that the Named Graph’s GUPRI can be used in the content’s place^69^. The unit’s contextual information, such as provenance, authorship, licensing, or the logical framework and data schema used, is stored in a meta-graph, linked via the unit’s GUPRI (Fig. 3a). In a relational or tabular database, content and metadata may be captured in separate tables associated with the same semantic unit class (Fig. 3b). Regardless of implementation, the *conceptual layering* ensures that semantic content and context remain independently addressable, enhancing clarity, reproducibility, and interoperability.

Semantic units also introduce structural layering at the level of the knowledge infrastructure as a whole. By distinguishing between a *content-layer* (holding the domain-specific statements) and a *meta-layer* (holding contextual information and metadata about units and their interrelations), knowledge infrastructures can maintain coherent semantic boundaries across both individual units and the system at large.

Semantic units are categorized into two top-level categories: statement units, which represent individual propositions or statements, and compound units, which aggregate multiple semantic units into coherent, internally consistent meaning carrying structures.

All semantic units guarantee four core properties: *internal semantic coherence*, *referability via a persistent identifier*, *annotatability* for provenance, context, or other metadata, and *decoupling of representation and documentation of meaning* for human-interpretability. They are *not merely documents, Named Graphs, or reified triples*, but conceptual primitives for structuring knowledge in a way that is both machine-actionable (FAIR) and human-comprehensible (CLEAR). Lightly, semantic units can represent assertions, observations, procedures, arguments, or other meaningful knowledge fragments, with the flexibility to compose more complex knowledge structures while maintaining the integrity of each unit.

Operational and implementation aspects of semantic units, including modelling workflows and integration within existing RDF/OWL and labeled property graph infrastructures, have been described in detail in the original semantic unit publication^69^. In the present paper, we therefore focus on the conceptual structure of semantic units and their role in addressing the representational challenges identified in the *Introduction*, rather than duplicating implementation-level material.

##### Statement unit

A statement unit captures the *smallest units of propositional meaning* within knowledge infrastructures. It corresponds to a single, coherent statement, such as an observation, measurement, classification, or relational claim, that can be treated as a first-class semantic object. When implemented in a knowledge graph, every triple in the graph belongs to exactly one statement unit, so that the set of all statement units forms a *mathematical partition* of the graph’s content-layer. The content-layer of a knowledge graph can thus be understood as the union of all its statement unit content-graphs.

**Fig. 4:**
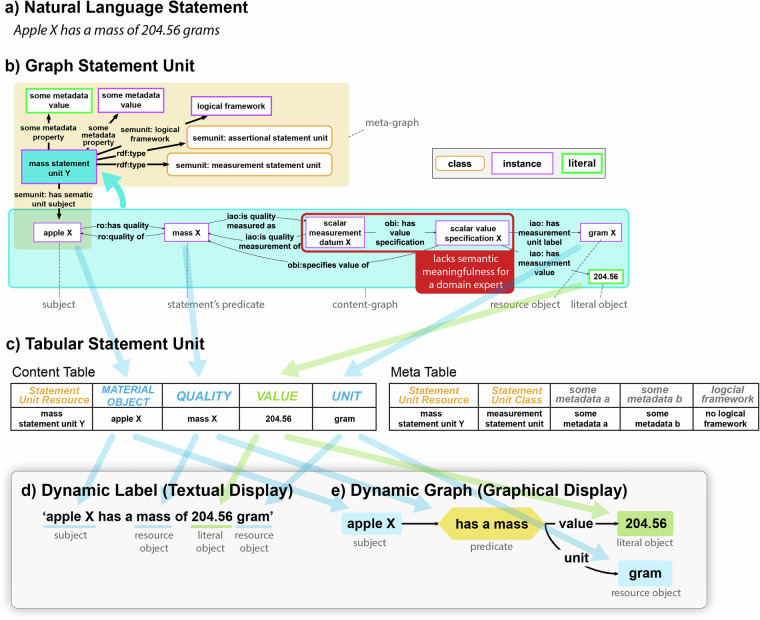
From natural language statement to graph and tabular statement unit. (**a**) A human-readable assertional statement about the mass measurement of an apple *X*. (**b**) The corresponding representation of the same statement as an instance-based semantic graph, organized within the content-graph (light-blue box with blue border) of a corresponding statement unit, adhering to RDF syntax and following the established pattern for measurement data from the Ontology of Biomedical Investigations (OBI)^100^. The content-graph articulates the statement with ‘apple X’ as its subject and ‘mass X’ and ‘gram X’ alongside the numerical value of 204.56 as its objects. The peach-colored box encompasses the unit’s meta-graph. It explicitly denotes the resource embodying the statement unit (blue box with purple border) as an instance of the *SEMUNIT:measurement statement unit* and the *SEMUNIT:assertional statement unit* class, with ‘apple X’ identified as the subject. Notably, the GUPRI of the statement unit is also the GUPRI of the semantic unit’s content-graph (indicated by the opaque blue arrow). The meta-graph also contains various metadata triples, here only indicated by *some metadata property* and *some metadata value* as their placeholders. Highlighted in red within the content-graph is an example of a triple that is required for modelling purposes but lacks semantic meaningfulness for a domain expert. (**c**) The same semantic content, modelled as a tabular statement unit. The measurement statement unit class has two tables associated, i.e., a content and a meta table. Any given statement unit instance of that type is documented by a row in each of these tables. (**d**) The dynamic label associated with the statement unit from b) and c). (**e**) The dynamic graph associated with the statement unit from b) and c). The transparent blue and green arrows indicate the mappings between slots in the graph schema, cells in the table, and slots in the dynamic label and dynamic graph, indicating semantic equivalence across the respective slots.

Each statement unit has a *subject resource* and one or more *object resources or literals*, reflecting the arity of the underlying predicate or verb (Fig. 4c). The number of constituent data points (e.g., triples in RDF-based implementations or cells in a table-row) of a statement unit therefore varies depending on the semantic complexity of the proposition being expressed. In contrast to atomic triple-based representations, statement units explicitly group all triples or cells in a row of a table that jointly express a single proposition, thereby preserving semantic coherence and mitigating fragmentation.

The content-graph or content-table row of a statement unit conforms to a corresponding *semantic schema*—a reusable template that specifies how a particular type of proposition is represented in the knowledge infrastructure. Such schemata may be implemented using technologies such as Shape Constraint Language (SHACL) shapes^71^ or Linked Data Modeling Language (LinkML) models for RDF/OWL, SQL Data Definition Language (SQL DDL) schemata^72,73^ for relational databases, or JSON Schema for JSON-based systems. Independent of the technical implementation, the schemata serve multiple purposes: they ensure representational consistency, enable validation, and support propositional interoperability^9^ across datasets and domains. By making the internal structure of statements explicit, semantic schemata also support systematic comparison and reuse of statements of the same type.

Beyond machine-actionability, statement units are designed to support *cognitive interoperability* (CLEAR) by providing a stable interface between formal representations and human interpretation. Because statement units explicitly identify subjects, objects, and their relationships, they can be rendered into human-facing representations without ad hoc interpretation logic. These *UI display patterns* could take the form of:

- **Dynamic labels**: human-readable, textual renderings derived from subject and object components of a statement unit, suitable for use in documentation, tables, or HTML-based interfaces (Fig. 4d).
- **Dynamic graph views**: structured visualizations, such as mind-map-like graphs, that represent the same semantic content in a form optimized for exploration and sense-making by human users (Fig. 4e).

These representations do not introduce new semantics, but rather expose the already formalized propositional structure in cognitively accessible forms, thereby addressing the CLEAR requirement that knowledge be interpretable and explorable by humans as well as machines^5^. A given statement unit type can have several such representational display patterns defined that can be used context-dependently, showing for instance only parts of the available information to a lay person while providing the full richness of information to an expert.

Because statement units constitute the smallest carriers of semantically meaningful content, they also serve as the primary anchoring points for semantic metadata, including authorship, provenance, which logical frameworks were applied for modelling and interpreting the content, and specifications of confidence or evidence levels. Attaching such metadata at the level of individual propositions supports traceability, reuse, and controlled reasoning and thus key requirements for FAIR and CLEAR knowledge infrastructures.

**Fig. 5:**
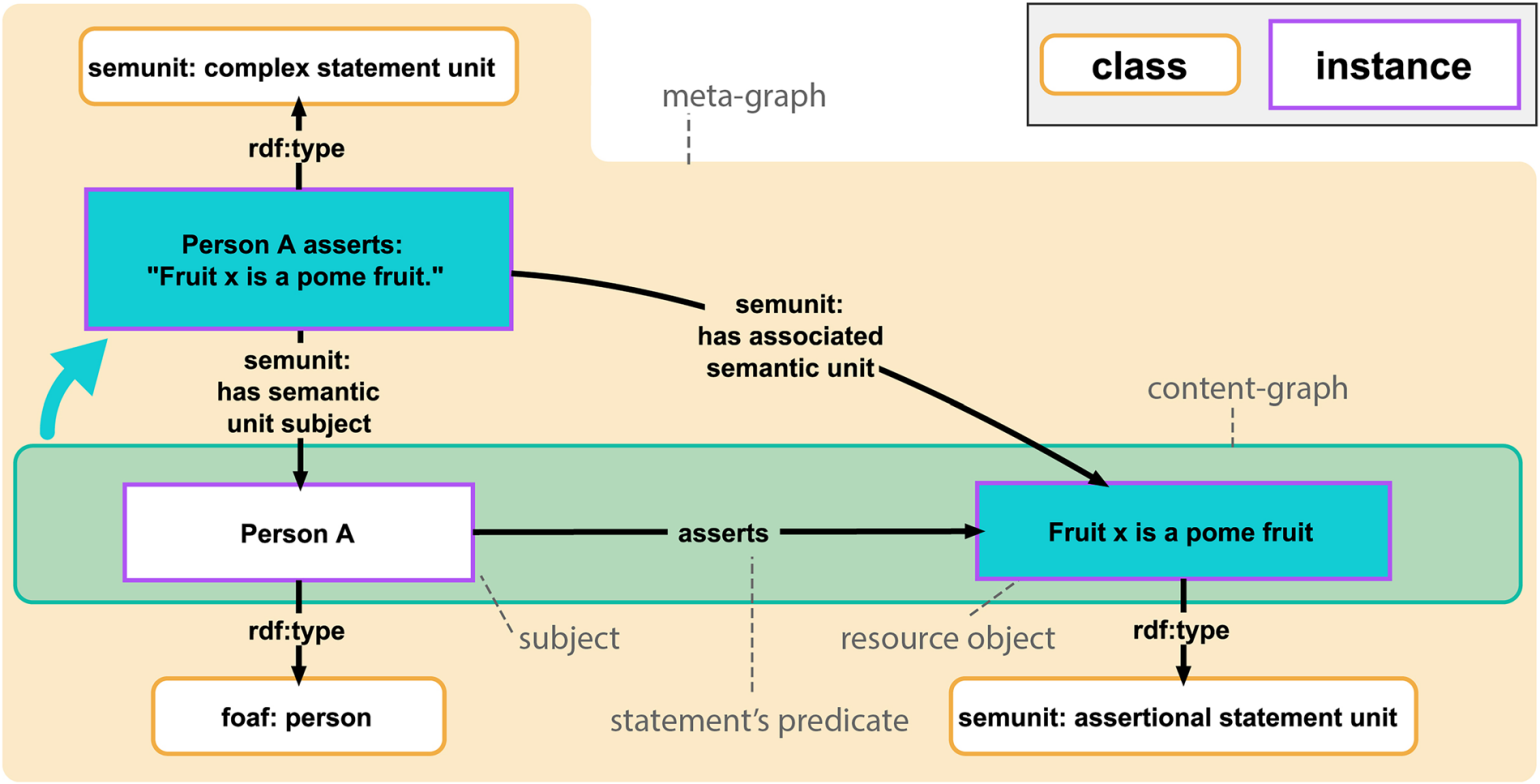
Example of an RDF-based complex statement unit in which at least one semantic unit resource takes the subject or one of the object positions of the statement. Here,’Person A’ (FOAF:person) takes the subject position and the assertional statement unit resource *’Fruit X is a pome fruit’* the object position of a statement, forming the content-graph of the complex statement unit. *Here, only the graph implementation is shown*.

To enable statements *about* statements—such as quotation, attribution, qualification, or expression of relationships between statements—we introduce *complex statement units*. Complex statement units combine the characteristics of statement units with those of higher-order semantic units: they possess their own content-graph or table while referencing one or more other statement units via the property *SEMUNIT: has associated semantic unit* (Fig. 5) or a corresponding table column. Complex statement units are modelled as a subclass of statement units, allowing statements themselves to occupy subject or object positions in higher-order propositions.

By treating statements as first-class semantic objects with explicit internal structure and identity, statement units directly address several challenges identified in the challenges sections above, including the representation of non-asserted content, epistemic attribution, disagreement, and the controlled composition of meaning. At the same time, statement units deliberately remain minimal. They capture single propositions but do not themselves impose aggregation, sequencing, or contextual bundling. These higher-level organizational concerns are addressed by *compound units*.

##### Compound unit

**Fig. 6:**
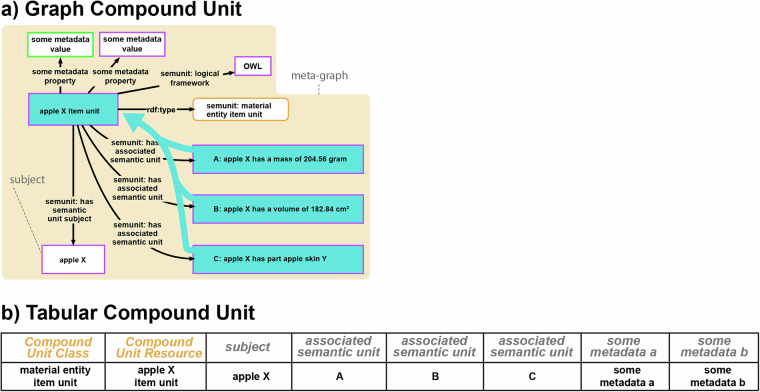
Example of a compound unit, denoted as *’apple X item unit’*. (**a**) The RDF-based compound unit. (**b**) A semantically equivalent implementation as tabular compound unit. Either version encompasses multiple statement units describing apple X (statement units *A*, *B*, and *C*). Compound units, by virtue of merging the content-graphs or content tables of their associated statement units, only indirectly manifest a content-graph or table. Consequently, compound units possess only a meta-graph (depicted in the peach-colored box) or meta table, referencing the associated semantic units.

A compound unit serves as a higher-level organizational resource that groups a *semantically meaningful collection of semantic units* (statement units and compound units). Each compound unit is identified by its own GUPRI and instantiates a corresponding compound unit class (Fig. 6).

Unlike statement units, compound units do *not* directly carry semantic content and therefore do not contain a content-graph or content table. Their meaning is derived entirely from the semantic units they reference. As a result, compound units contribute exclusively to the meta-layer of a knowledge infrastructure, where they establish structure and contextual coherence without introducing additional assertions.

#### Instance-quantified resource categories

The OWL framework distinguishes three fundamental categories of representational resources: *named-individuals* (particulars), *classes* (universals or kinds), and *properties* (relations). While this taxonomy provides a solid foundation for ontology modelling, and OWL and related formalisms are logically expressive enough to encode existential and universal constraints at the class level, these representational categories are insufficient for representing such statements as first-class, referable knowledge elements. As discussed in the challenges sections above, existential and universal claims are typically relegated to anonymous class axioms and blank nodes, which cannot be directly identified, attributed, versioned, or contextualized. Prototypical statements are not supported at all within standard Description Logics. Consequently, although these statements can be encoded indirectly, they cannot be represented as explicit, instance-level resources that support FAIR and CLEAR Principles. This motivates the introduction of four additional *instance-quantified resource categories* that extend OWL’s representational vocabulary at the level of referable resources rather than logical operators.

Importantly, these categories are *representational constructs*, not extensions of OWL’s formal semantics. When implemented in RDF/OWL, they behave as ordinary individuals from the perspective of standard reasoners. The limitation addressed here is therefore not one of formal logic, but of representational granularity and epistemic accessibility. The additional resource categories make quantified scopes explicit, referenceable, and annotatable within a knowledge infrastructure, without presupposing automated logical inference.

##### Some-instance resource

A *some-instance resource* denotes one or more unspecified instances of a target class *C*. It is used to represent existential statements of the form: “*There exists at least one instance of C such that…*”.

Formally, this can be notated as: ∃ *i* *∈* *C*, where *i* denotes an instance and *C* the class.

A some-instance resource is typed both as an instance of *SEMUNIT: some-instance resource* and as an instance of the target class *C*. When used as a subject or object of a statement unit, it indicates that the statement applies to an implicit, non-empty subset of instances of *C*, without enumerating or identifying them individually.

This resource category is particularly useful in contexts where assertional statements about specific individuals are inappropriate or impossible, such as for the representation of portions of matter. To illustrate, a particular portion of water can be represented by associating an ‘object aggregate’ (BFO:0000027) with a some-instance resource of water (CHEBI:15377) through *hasPart* (BFO:0000051), without committing to the identity of a specific instance of a water molecule.

##### Most-instances resource

A *most-instances resource* represents a typical but not universal subset of instances of class *C*, thus providing prototypical or majority-based generalizations over *C*. A most-instances resource is used to express statements such as “*Most birds fly*”, where a property holds for a typical subset of instances but admits well-known exceptions (e.g., ostriches, penguins).

Formally, a most-instances resource denotes a subset *D* *⊆* *C* such that the cardinality of *D* exceeds that of its complement within *C*. The resource is typed both as an instance of *SEMUNIT: most-instances resource* and as an instance of the target class *C*.

By making such prototypical claims explicit and referable, most-instances resources enable the representation of default expectations and typical properties without asserting incorrect universal claims.

##### Every-instance resource

An *every-instance resource* expresses distributive universal quantification over a class *C*. It corresponds to statements of the form “*For every instance i of C, it holds that…*”.

Formally, this can be notated as: ∀ *i* *∈* *C.*

An every-instance resource is typed as an instance of *SEMUNIT: every-instance resource* and as an instance of the target class *C*. It is intended to represent strict, logically universal claims that apply individually to each member of a class, such as definitional or law-like statements (e.g., “*Every instance of water consists of hydrogen and oxygen atoms”*).

##### All-instances resource

An *all-instances resource* denotes the entire population of instances of a class *C* considered collectively, across all time (past and present). It is used for population-level, statistical, or comparative statements that do not distribute over individuals, such as comparing cardinalities (e.g., *“There are more prokaryotes than eukaryotes”*) and expressing distributional properties (e.g., “*The average height of adult humans is…*”).

An all-instances resource is typed both as an instance of *SEMUNIT: all-instances resource* and as an instance of the target class *C*. In contrast to every-instance resources, which support distributive universality, all-instances resources support collective reference to a class’s extension across time.

When combined with semantic units, these instance-quantified resource categories enable the explicit representation of existential, prototypical, distributive, and collective statements as first-class referable elements of a knowledge infrastructure. Rather than encoding such statements implicitly in logical axioms, they can be documented, contextualized, and compared in ways that support both FAIR machine-actionability and CLEAR human interpretability. Section '*Addressing the challenges using the Semantic Units Framework*' demonstrates how the resulting Semantic Units Framework addresses the conceptual challenges identified in Section '*Conceptual challenges in FAIR and CLEAR data and knowledge infrastructures*'.

#### Relation to other modularization and abstraction approaches

Several existing frameworks address statement-level annotation, provenance, or modularization in knowledge graphs, most notably nanopublications and RDF-star. Nanopublications^50–53^, building on Named Graphs^47^, provide a well-established mechanism for packaging an assertion with its provenance and publication metadata into a single identifiable artefact. They have proven to be a viable implementation substrate for semantic units. In fact, the work that first introduced the semantic unit concept explicitly discusses how nanopublications can be used to implement, publish, and exchange semantic units across distributed systems^69^.

While nanopublications focus on the publication and provenance of individual assertions represented as RDF triples, the Semantic Units Framework operates at a more abstract and technology-agnostic representational level. It defines semantic units as first-class *semantic objects* with explicit internal structure, identity, and epistemic status, encompassing not only individual propositions but also high-order aggregations and contextual groupings. From this perspective, nanopublications are not an alternative to semantic units but one possible realization mechanism for specific types of semantic units.

RDF-star^48,74,75^ introduces quoted triples as a syntax shorthand for making statements about single triples, facilitating annotation and provenance tracking within RDF stores. While useful for certain annotation tasks, RDF-star remains fundamentally triple-centric and does not, by itself, provide abstractions for grouping multi-triple propositions, representing quantification, managing epistemic status, or defining higher-level compositional structures. Semantic units, by contrast, are defined independently of any specific graph syntax and can be mapped onto RDF-star for simple cases, while extending beyond it in cases where semantic coherence requires more than a single quoted triple. Rather than competing with RDF-star, the Semantic Units Framework subsumes it as a low-level realization option within a broader, technology-agnostic representational model.

Beyond statement annotation, several approaches have been proposed to extract or approximate semantically meaningful substructures from RDF graphs, including graph kernel methods for RDF data^76^, rooted graph abstractions and least common subsumers^77^, and query tree–based interaction models^78^. These approaches primarily serve analytical or computational purposes, and typically derive graph fragments dynamically from an existing knowledge graph. Semantic units differ fundamentally in intent and function, as they are not analytical artefacts computed from graphs, but representational primitives used to construct graphs from the outset. Semantic units define persistent, identifiable boundaries of meaning, explicitly encoding semantic scope, epistemic status, and contextual coherence.

Accordingly, these approaches are best understood as complementary rather than competing. Semantic units can provide stable, semantically coherent inputs to kernel-based analysis, rooted graph comparison, or query-generation techniques, while addressing representational challenges, such as non-asserted content, epistemic attribution, semantic fragmentation, and identity of granular entities, that lie outside the scope of extraction- and analysis-oriented methods.

While the Semantic Units Framework is presented at a conceptual and representational level, it is designed to be directly operationalizable using existing Semantic Web and data modelling technologies. Statement units and compound units can be implemented using RDF Named Graphs, relational tables, or JSON-based representations, with semantic schemata implemented via SHACL shapes, LinkML models, SQL DDL or equivalent schema languages. Transformation rules described in this paper correspond to deterministic restructuring operations and can be expressed using standard mechanisms such as SPARQL CONSTRUCT queries, SHACL rules, or ETL pipelines. A reference realization of Semantic Units based on nanopublications and RDF-based tooling has been discussed in prior work^69^, demonstrating the practical feasibility of the framework for publishing, versioning, and provenance-aware knowledge representation. The following section builds on this foundation by illustrating how these representational constructs address the challenges identified above, when combined with instance-quantified resource categories.

### Addressing the challenges using the semantic units framework

This section shows how the Semantic Units Framework addresses the representational challenges identified above. To maintain structural correspondence, the section is organized into two subsections that mirror the earlier analysis: Subsection '*Addressing paradigmatic OWL expressivity limitations*' addresses the challenges discussed in Section '*OWL Expressivity as a paradigmatic challenge*', where OWL expressivity served as a paradigmatic reference point, while Subsection '*Addressing general challenges of meaning, epistemics, and identity*' addresses the more general challenges of meaning, epistemics, fragmentation, and identity introduced in Section '*From OWL limitations to general challenges of representing meaning, epistemics, and identity*'.

**The solutions are presented at a conceptual and representational level. For reasons of clarity and concreteness, illustrative examples are shown using RDF-based realizations of semantic units. These examples are intended to demonstrate feasibility and expressive power, not to prescribe a specific technology stack. The underlying solutions are independent of RDF/OWL and can be realized using alternative graph, relational, or JSON-based infrastructures**.

#### Addressing paradigmatic OWL expressivity limitations

This section demonstrates how the Semantic Units Framework, together with the instance-quantified resource categories introduced above, resolves the expressivity challenges identified in Sections '*Foundational challenge: Unified representation of categories of statements*' and '*Challenge: Representing universal statement*' by enabling explicit, instance-based representations of diverse statement types within a knowledge graph.

##### Five categories of statement units for expressive knowledge representation

**Lexical statement unit**: Lexical statements units play a crucial role in reducing cognitive fragmentation by making the *representational status* and *linguistic grounding* of resources explicit and machine-readable. They capture statements about labels, synonyms, usage examples, elucidations, and other linguistic or editorial annotations associated with resources in a knowledge graph.

A central subclass of lexical statement units is the *identification unit*, which specifies *to what kind of representational resource category* a given GUPRI belongs. Although such information could be viewed as ontological rather than lexical in nature, it is treated here as part of the lexical layer for pragmatic reasons, because identification units provide the interpretive context required to correctly read, display, and manipulate resources within semantic units.

**Fig. 7:**
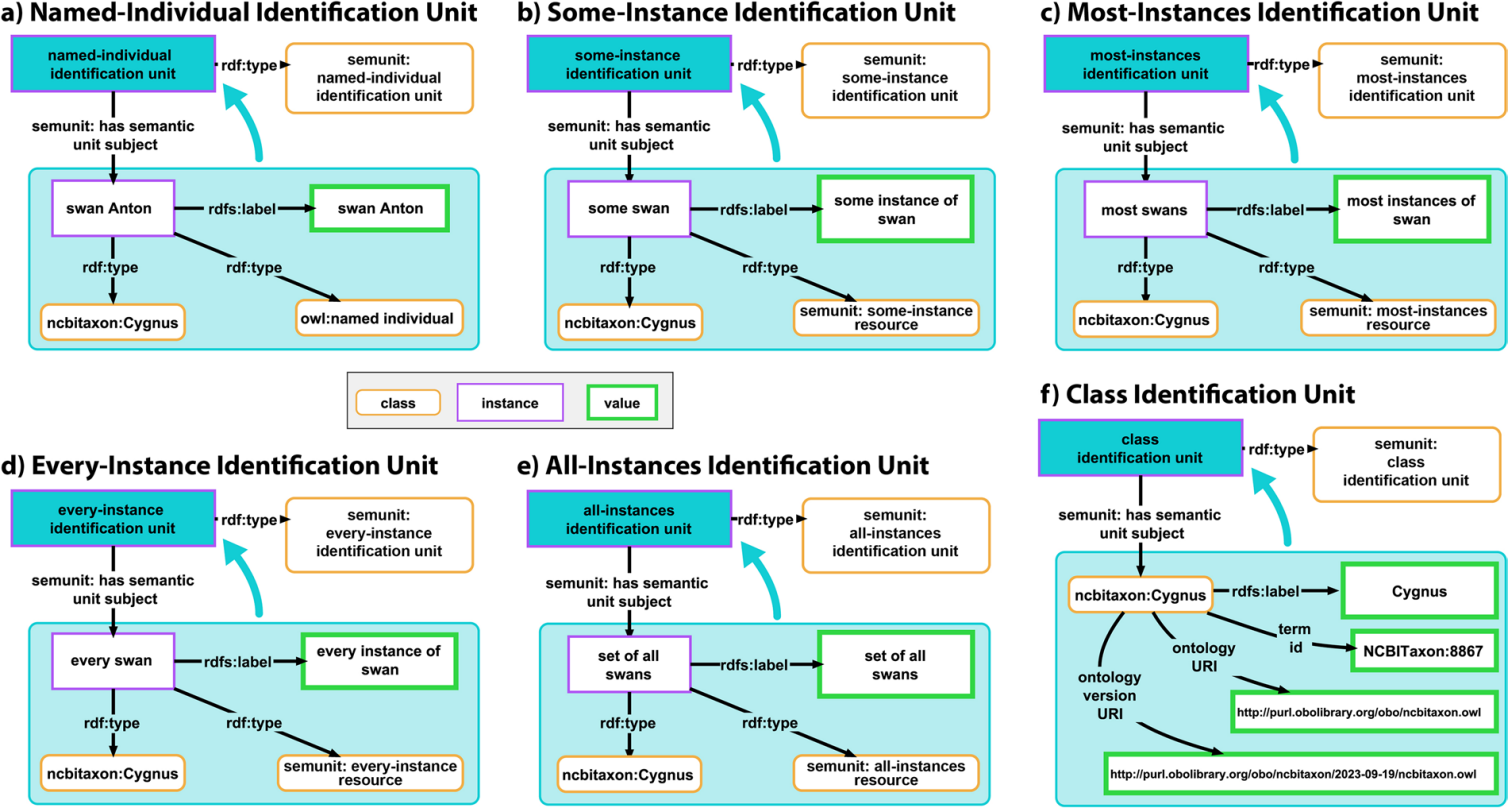
Examples for six different types of identification units. They are distinguished based on the category of their subject resource. Each identification unit is represented by its own unit resource (blue box with purple border), with its content-graph shown by the light-blue box. Except for the class identification unit (f), all subject resources of identification units are specified through the property *type* (RDF:type) as instances of two distinct classes, one of which is the category of (instance-quantified) resource and the other one the type of entity (here, the swan Cygnus (NCBITaxon:8867)). (**a**) A *named-individual identification unit*. (**b**) A *some-instance identification unit*. (**c**) A *most-instances identification unit*. (**d**) An *every-instance identification unit*. (**e**) An *all-instances identification unit*. (**f**) A *class identification unit* that denotes the label and the identifier of the class Cygnus (NCBITaxon:8867). Optionally, it also provides the URI of the ontology and that of the ontology version from which the class was taken. *For reasons of clarity, metadata for each identification unit is not represented*.
- *Some-instance*, *most-instances*, *every-instance*, and *all-instances identification units* identify a resource as an instance-quantified representative of a target class, differing in scope or quantification (e.g., existential, prototypical, universal, or population-level)(Fig. 7b-e).
- *Class identification units* reference ontology classes and typically include metadata such as the class label, class GUPRI, the GUPRI of the source ontology, and the ontology version (Fig. 7f).

We distinguish six forms of identification units, each corresponding to a specific resource category:

- *Named-individual identification units* identify a resource as a named-individual resource of a particular target class and associate it with a human-interpretable label (Fig. 7a).

Analogous identification units could also be defined for properties. However, the present work focuses on resources that function as subjects or objects of statement units.

By explicitly encoding the representational and linguistic context of resources, lexical statement units stabilize interpretation, reduce implicit assumptions about resource identity, and support cognitively accessible renderings of semantic units. In this way, they provide a foundational layer that enables the expressive use of assertional, existential, prototypical, and universal statement units within a unified semantic framework (see next subsections).

**Assertional statement unit**: Assertional statement units express propositions about *named individuals* and correspond to classical ABox assertions in OWL, typed as both *SEMUNIT: assertional statement unit* and as a relation-based statement unit class (cf. statement unit classification in^69^). When a statement includes an object resource, this resource must also be a named individual.

**Fig. 8:**
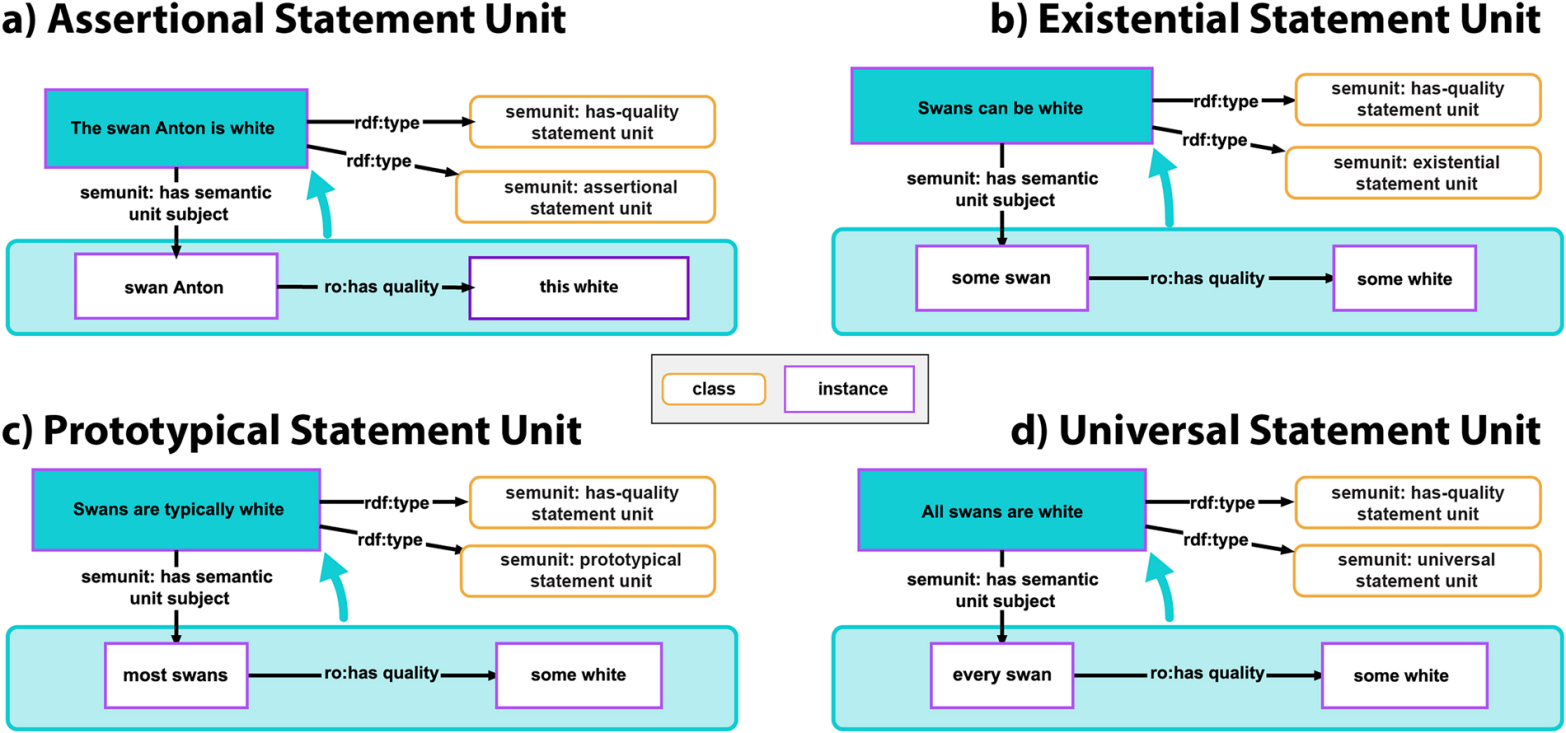
Four basic categories of statement unit. All four examples model a *has-quality* relation between two resources and differ in terms of which categories these two resources belong to. Their content-graphs (light-blue boxes) express this relation as an instance-based ABox graph. Their unit resource (blue box with purple border, here shown with their dynamic label) always instantiates two classes, indicating the type of relation (*SEMUNIT: has-quality statement unit*) and the basic statement category. (**a**) This *assertional statement unit* models the relation between two named individuals. Its subject is a resource that is also the subject of a named-individual identification unit (cf. Figure 7a). (**b**) This *existential statement unit* models the relation between some instance of ‘swan’ (NCBITaxon:8867) and some instance of ‘white’ (PATO:0000323). Its subject is thus also the subject of a some-instance identification unit (cf. Figure 7b). (**c**) This *prototypical statement unit* models the relation between most instances of ‘swan’ (NCBITaxon:8867) and some instance of ‘white’ (PATO:0000323). Therefore, its subject is also the subject of a most-instances identification unit (cf. Figure 7c). (**d**) This *universal statement unit* models the relation between every instance of ‘swan’ (NCBITaxon:8867) and some, but not necessarily every, instance of ‘white’ (PATO:0000323). Its content-graph can, in principle, be translated into an OWL TBox graph. Its subject is also the subject of an every-instance identification unit (cf. Figure 7d). *For reasons of clarity, metadata for each semantic unit is not represented*.

Figure 8a shows an example in which the subject is the named-individual resource ‘swan Anton’ (NCBITaxon:8867). Assertional statement units are thus well-suited for expressing factual claims about known, context-specific entities.

**Existential statement unit**: Existential statement units express *existential claims* involving *anonymous instances* of a target class, represented as *some-instance resources*. They capture propositions that hold for at least one instance of a given class, supporting knowledge derived from empirical or partial evidence, such as observations of possibilities, rather than universal truths. When a statement includes an object resource, this object must also be a *some-instance resource* (Fig. 8b). Each existential statement unit is typed both as *SEMUNIT: existential statement unit* and as a statement unit class based on the underlying relation of the statement (*has-quality* in the examples in Fig. 8).

**Prototypical statement unit**: Prototypical statement units represent *typical, but not universal*, characteristics of instances of a target class *C*. They use a *most-instances* resource as their subject, enabling the representation of *prototypical relationships* and thus general tendencies, empirical regularities, and default relationships, such as common disease symptoms or typical drug effects, that admit exceptions (see also Fig. 8c).

Because OWL and Description Logics are strictly monotonic, such prototypical statements cannot be represented as axioms without loss of meaning—*OWL and Description Logics do not natively support the notion of prototypicality*. To provide formal semantics^29^, we define a subclass *D* of the target class *C*, whose instances share a property set *A*. A prototypical statement is considered valid if the cardinality of *D* exceeds that of the complement set (|*D*| > |*C* \ *D*|), corresponding to interpretations such as “*Most apples are green*,” meaning that green apples outnumber non-green ones within the class apple.

This approach enables the structured representation of prototypical knowledge while preserving the possibility of exceptions without resulting in logical inconsistencies with the knowledge infrastructure.

**Universal statement unit**: Universal statement units express *generalizations that necessarily hold for all instances* of a given class. The subject is either the class itself or a quantified universal resource (*every-instance* or *all-instances*). Two structural patterns are distinguished:

- *Class-based universal statements* use OWL constructs such as *subClassOf* (RDFS:subClassOf), *equivalentClass* (owl:equivalentClass), *disjointWith* (owl:disjointWith), or *sameAs* (owl:sameAs) to express relationships between classes. When OWL properties are interpreted as universals, analogous patterns apply to properties using constructs such as *subPropertyOf* (owl:subPropertyOf), *domain* (owl:domain), or *range* (owl:range).
- *Quantified universal statements* use an *every-instance* or *all-instances* resource as their subject. When such statements include an object resource, it is typically a *some-instance* resource, resulting in an *all-to-some* relation. In n-ary statements, multiple object resources may be involved.

For example, the statement “Every swan (NCBITaxon:8867) *hasQuality* (RO:0000086) some white (PATO:0000323)” is represented as a universal statement unit with an *every-instance* subject and a *some-instance* object (Fig. 8d). Universal statement units thus support the formalization of domain axioms and universally valid rules as instance-based ABox graphs or tables within a knowledge infrastructure.

##### Formal semantics and reasoning implications

As discussed in Section '*OWL Expressivity as a paradigmatic challenge*', OWL’s native semantics primarily support assertional and universal statements, while existential and prototypical statements lack adequate formal treatment within strictly monotonic Description Logics. The Semantic Units Framework extends the representational scope of OWL-based knowledge graphs by introducing statement-level abstractions that make such distinctions explicit, while remaining compatible with existing ontology infrastructures.

Semantic units elevate statements to first-class, identifiable entities in the knowledge graph. Each unit constitutes a self-contained semantic module that can be independently annotated, contextualized, queried, and, where appropriate, associated with a formal interpretation. This modularization enables individual statements, including universal claims and class-level axioms, to be referenced and discussed within the general domain of discourse, directly addressing the challenges of semantic fragmentation and implicit context identified in the challenges sections.

From a formal perspective, statement units can be understood as mediators between instance-level representations and logical formalisms. Depending on their category, they may correspond to classical ABox assertions, to TBox-style axioms expressed via ontology design patterns^79^, or to rules in non-monotonic or rule-based formalisms. Prior work^80^ has shown how such correspondences can be defined using relational ontology design patterns or logic programming rules, enabling translations between instance-based representations and formal axioms where required. Importantly, this interpretive layer may be explicit and external to the graph itself, allowing reasoning strategies to be selected according to the nature of the statement.

This flexibility is essential for handling non-monotonic knowledge, such as defaults, exceptions, and prototypical statements, which cannot be faithfully represented within OWL’s monotonic semantics. Rather than forcing such statements into inappropriate universal axioms, semantic units allow them to be represented under explicit formal assumptions, including rule-based or defeasible semantics, without compromising the integrity of the overall knowledge graph.

More generally, the modular structure of the Semantic Units Framework allows a single knowledge graph to integrate data and knowledge modelled under different logical or rule-based frameworks. Each semantic unit can indicate within its meta-graph the formal setting under which its content is intended to be interpreted, avoiding the need for a single global logic. By contrast, restricting a knowledge graph to one logical framework, such as Description Logics as realized in OWL, necessarily excludes or distorts certain statement categories, leading to loss of meaning, unintended reinterpretations, or reliance on informal modelling conventions. Semantic units address this limitation by decoupling representation from formal interpretation, enabling heterogeneous but explicitly grounded reasoning within a unified semantic structure.

##### Addressing complex class axioms

To address the challenge of representing complex class axioms, particularly those involving chained or triangular relationships (see Section '*Challenge: Representing complex class axioms*'), we introduce a representational strategy based on three subcategories of compound units: *item units*, *item group units*, and *class profile units*. Together, these units enable the expression of class axioms within the ABox using instance-quantified resources rather than blank nodes, while remaining compatible with OWL semantics via explicit translation patterns^80^.

**Universal item unit**: As discussed in previous work^69^, an item unit is a compound unit that aggregates all statement units sharing the same subject resource. Depending on the subject resource category, item units may be assertional (named-individuals), existential (some-instance), prototypical (most-instances), universal (every-instance), or all-instances item units.

**Fig. 9:**
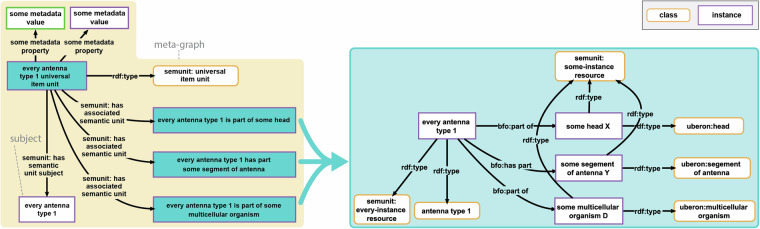
Universal item unit. **Left:** An example of a universal item unit, with three associated universal statement units. **Right:** The content-graph of the same universal item unit, resulting from merging the content-graphs of the three associated universal statement units. Each statement unit has *‘every antenna type 1’* as their subject. *For reasons of clarity, neither resources and relations of associated semantic units nor their metadata shown*.

*Universal item units* can be used to represent simple, *star-shaped class axioms* by specifying properties that necessarily hold of every instance of a given class (Fig. 9). When such a unit captures *sufficient conditions* for class membership, it constitutes a *sufficient universal item unit*.

**Universal item group unit**: An item group unit is a compound unit formed by two or more item units whose content-graphs are interconnected through intermediate statement units, such that the object of one statement becomes the subject of another^69^. This structure enables the explicit representation of *relational chains* and *triangular patterns* when applied to modelling corresponding class axioms.

**Fig. 10:**
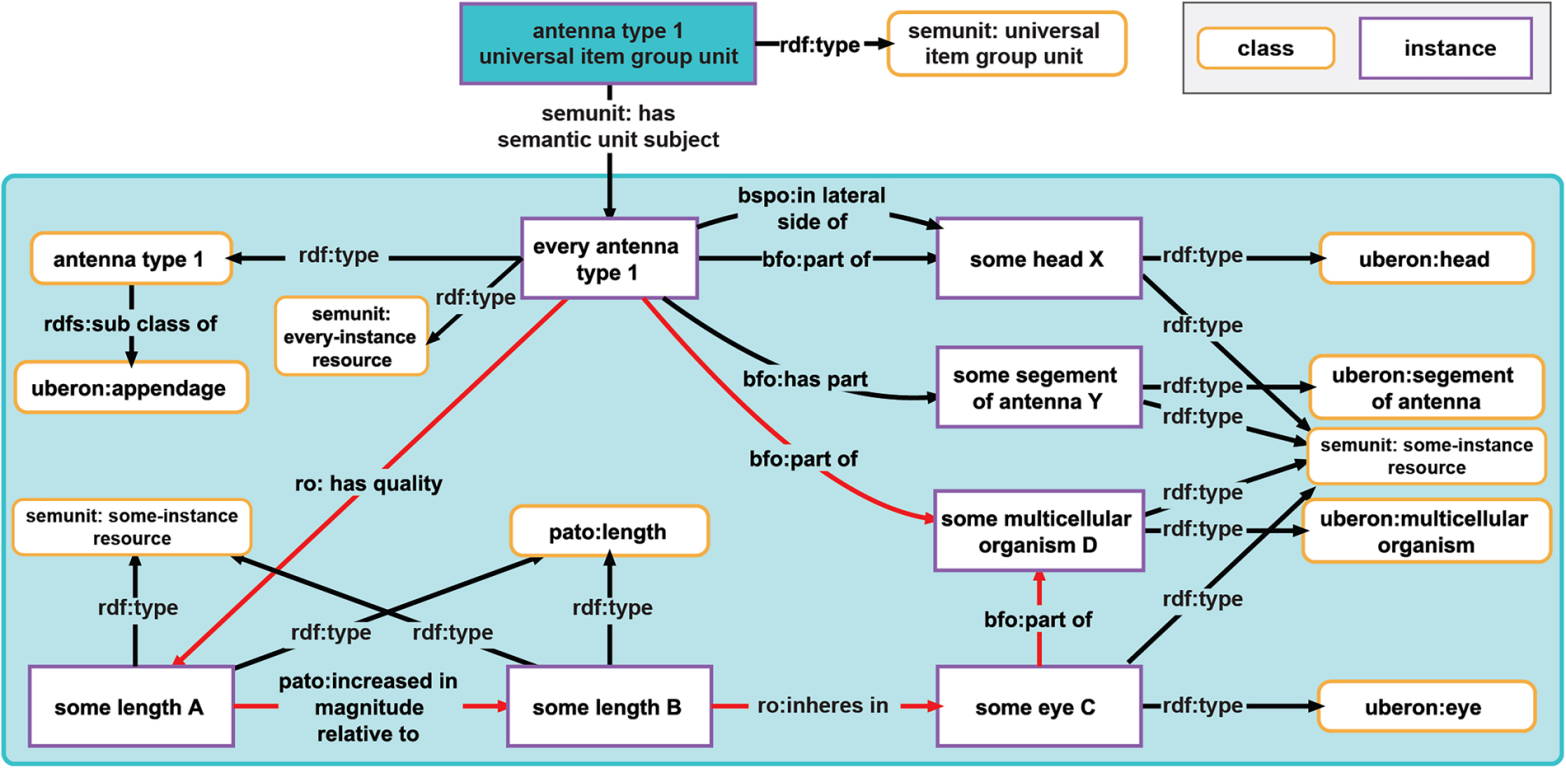
Class axiom expressed as a universal item group unit. A universal item group unit that corresponds with the Manchester Syntax expression of the class axiom for *antenna type 1*, where multicellular organism (UBERON:0000468) is referenced twice, resulting in two independent blank nodes when turned into an OWL TBox expression (see Section '*Challenge: Representing complex class axioms*'). Its content-graph is shown in the light-blue box (merged from the content-graphs of all associated item and statement units). The statements cannot be modelled in a single universal item unit, because they do not all share the same subject. Contrary to its corresponding OWL TBox expression, this notation allows describing triangular relations as the one shown here in red, indicating that every instance of *antenna type 1* is longer than any eye that is part of the same organism. See also the correlation between the use of “some” in Manchester Syntax in Section ‘*Challenge: Representing complex class axioms*’ and the use of corresponding some-instance resources in the universal item group unit. *For reasons of clarity, neither resources and relations of associated semantic units nor their metadata are shown*.

*Universal item group units* are required when class axioms cannot be expressed using a single universal item unit, for example when involving combinations of universal and existential constraints across multiple entities (Fig. 10; cf. with Manchester Syntax expression in Section '*Challenge: Representing complex class axioms*'). By using instance-quantified resources instead of blank nodes, the same anonymous entity can be consistently referenced across different parts of the axiom, preserving identity and intended semantics.

When a universal item group unit specifies sufficient properties for a class, it is a *sufficient universal item group unit*.

This approach avoids well-known OWL restrictions in representing triangular relationships while still allowing translation into OWL-compatible patterns for inference when combined with other OWL axioms^80^.

**Class profile unit:** While universal item units and item group units capture necessary and sufficient conditions for class membership, many real-world entity kinds are also characterized by *existential*, *prototypical*, or *probabilistic properties*. To represent this broader epistemic context, we define the *class profile unit*.

A class profile unit aggregates all universal, existential, and prototypical item (group) units associated with a class, providing a structured and comprehensive representation of both definitional and empirical diagnostic knowledge. This includes typical features, statistical tendencies, and diagnostic criteria that are informative for recognition and interpretation but not strictly required for class membership^33^.

For example, while a universal item group unit may define *antenna type 1* by a necessary anatomical relationship, its class profile may additionally record typical coloration or frequent occurrence in aquatic insects, capturing domain knowledge that would otherwise remain implicit or informal.

##### Addressing negations and cardinality restrictions

Negations and cardinality restrictions pose well-known modelling challenges in OWL, as both are primarily expressed through class-level constructs that are difficult to construct, query, and interpret, particularly when applied to instance-level knowledge (see Section '*Challenge: Representing negations and cardinality restrictions*'). To address this challenge, the Semantic Units Framework includes two new types of units: *negation units* and *cardinality restriction units*. These units enable negation and quantitative constraints to be represented explicitly in the ABox, while remaining translatable into OWL TBox axioms when required.

**Fig. 11:**
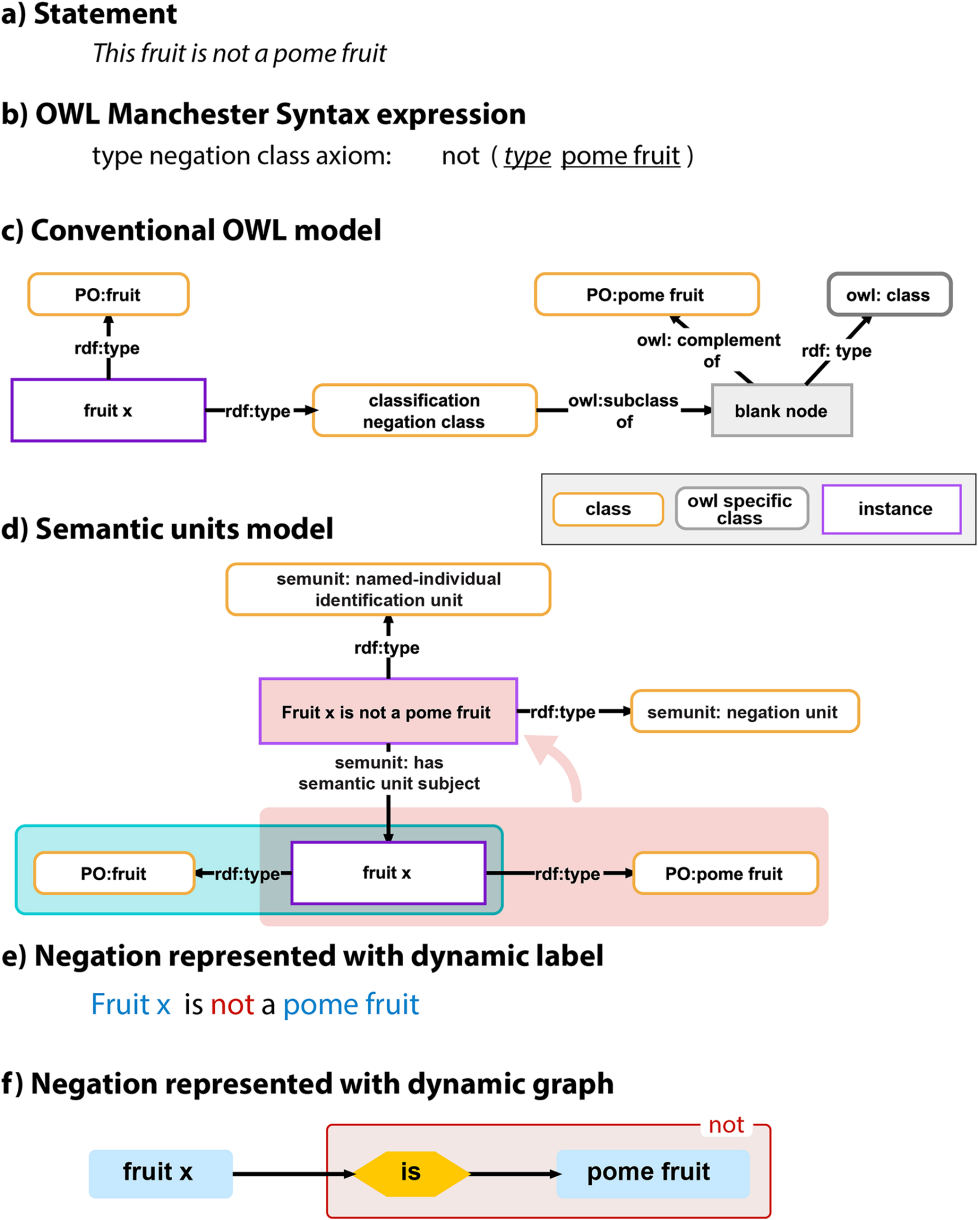
Representing negations within the Semantic Units Framework. (**a**) A human-readable statement that this fruit is not a pome fruit. The statement can be modelled in two different ways: As an OWL expression that can be specified using (**b**) Manchester Syntax, where the fruit is an instance of a class of which all instances are not instances of pome fruit (PO:0030110). Note, how this Manchester expression translates into (**c**) an OWL TBox expression mapped to RDF, where ‘fruit x’ is an instance of fruit (PO:0009001) but also of a class that is the complement to pome fruit (PO:0030110). Alternatively, the statement can be modelled as an instance-based ABox graph within the Semantic Units Framework using two statement units. (**d**) The content-graph in the light-blue box states that the entity is a fruit. The content-graph belonging to the negation unit (red box), on the other hand, states that the entity is a pome fruit. However, since the latter statement unit instantiates both *SEMUNIT: named-individual identification unit* and *SEMUNIT: negation unit*, it negates its content-graph, expressing that it is *not* a pome fruit. Based on UI display patterns, the unit’s semantic content can be displayed in a human-interpretable form, either as text through its dynamic label **e)** or as a graph through its dynamic graph **f)**. *For reasons of clarity, metadata for each semantic unit is not represented*.

**Negation units**: In OWL, negation is typically expressed via class complements. For example, the statement “*This fruit is not a pome fruit*” requires asserting membership in the complement of the class pome fruit (PO:0030110), which implicitly shifts the statement to the TBox level (Fig. 11a–c).

Within the Semantic Units Framework, the same meaning is represented more directly by classifying a statement unit as an instance of the *SEMUNIT: negation unit* (see Fig. 11d). The content-graph or table of the statement retains its original structure (e.g., *type* or *part-of*), by itself assuming the truth of the corresponding content,but its semantic polarity is inverted by this classification, consequently negating the unit’s semantic content. This allows negated assertions to remain instance-based, identifiable, and queryable, without requiring class complement expressions. For UIs, display patterns can render such statements in human-interpretable forms, meeting the CLEAR Principle (Fig. 11e,f).

**Fig. 12:**
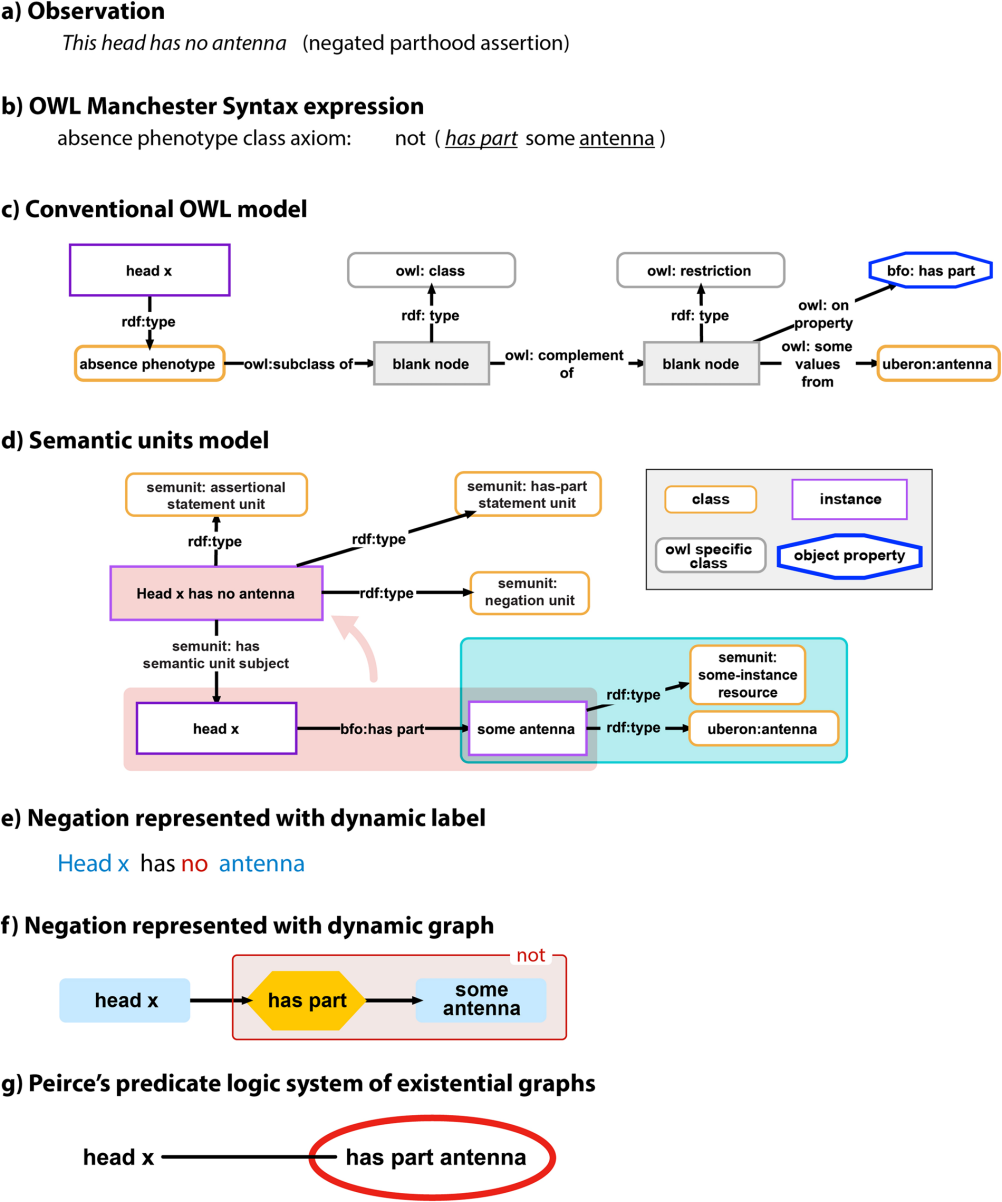
Representing absences within the Semantic Units Framework. (**a**) A human-readable statement about the observation that a given head has no antenna. In OWL, absence statements cannot be expressed as relations between instances but only as a class expression, which can be formulated using Manchester syntax (**b**). Following this notation, the head is an instance of a class whose members have no antenna as their parts (‘not’ and ‘some’ being used as mathematical expressions). (**c**) The translation of the assertion from a) and b) into an OWL TBox expression mapped to RDF. Note how *absence phenotype* is defined as a set of relations of subclass and complement restrictions involving two blank nodes. (**d**) The same statement represented within the Semantic Units Framework as an instance-based ABox graph using two semantic units. One semantic unit models the has-part relation and negates it (red box) and is typed as *SEMUNIT: has-part statement unit*, *SEMUNIT: assertional statement unit*, and *SEMUNIT: negation unit*. The other semantic unit is of type *SEMUNIT: some-instance identification unit*, with its content-graph shown in the light-blue box. Both units together model the observation from a). The semantic content of both units can be displayed in a human-interpretable form either as text through a dynamic label (**e**) or graphically through a dynamic graph (**f). (g**) An alternative notation of the statement ‘this head has no antenna’ and the observation from a). The notation uses Peirce’s predicate logic system of existential graphs. The *identity line* **―** between the two phrases ‘*head x*’ and ‘*has part antenna*’ states that head *x* has some antenna as its part, whereas the red circle surrounding the latter phrase expresses its negation by crossing the *line of identity*. *For reason of clarity, the relation between‘head x’ andhead (UBERON:0000033) is not shown in c) and d). Moreover, metadata for the semantic unit is not represented*.

**Fig. 13:**
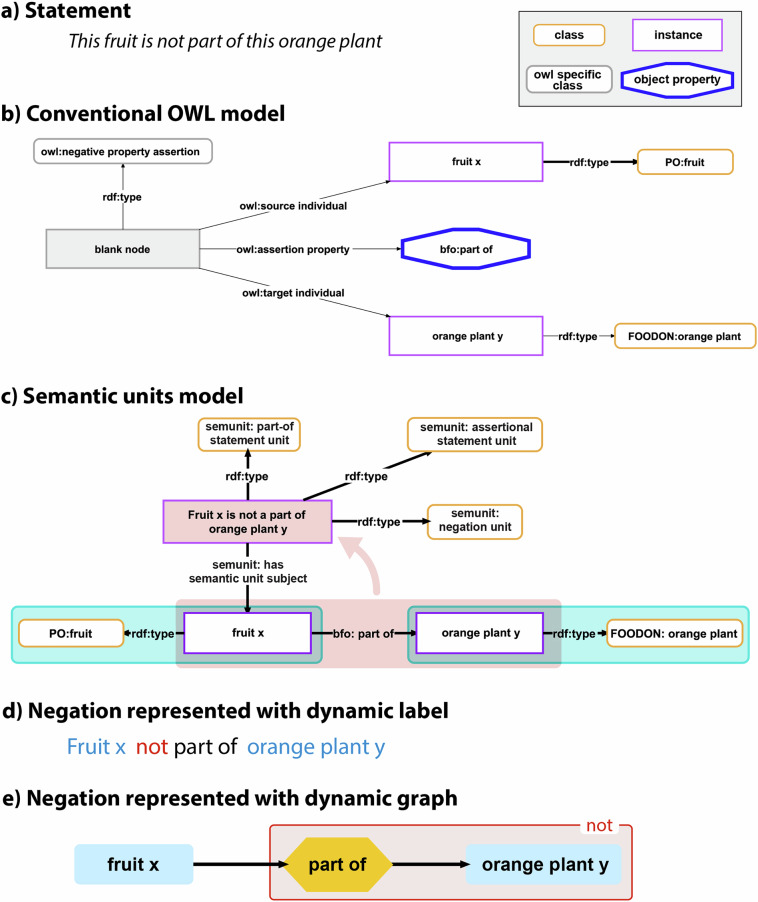
Representing negating relations between individuals within the Semantic Units Framework. (**a**) A human-readable statement that this fruit is not part of this orange plant. The statement can be modelled in two different ways: (**b**) as an OWL expression mapped to RDF. Note, how the statement is translated into a negated assertion statement with source, property, and target specification, relating an instance ‘fruit x’ (PO:0009001) to an instance ‘orange plant y’ (FOODON:03411339) involving a blank node. (**c**) Alternatively, the statement can be represented using three semantic units. The two named-individual identification units, with their content-graphs shown in the light-blue boxes, state that one of the objects is a fruit and the other one an orange plant, whereas the part-of statement unit (red box) states that fruit x is not part of orange plant y as the unit instantiates simultaneously *SEMUNIT: part-of statement unit*, *SEMUNIT: assertional statement unit*, and *SEMUNIT: negation unit*. The semantic content of the three statement units can be displayed either as text through a dynamic label (**d**) or graphically as a dynamic graph (**e**). *For reasons of clarity, metadata for the semantic unit is not represented*.

The same mechanism applies to absence statements (e.g., “*This head has no antenna*”, Fig. 12) as well as to negated relations between two specific individuals (e.g., “*This fruit is not part of this orange plant*”, Fig. 13), corresponding to OWL’s negative property assertions. In all cases, negation is modelled uniformly by typing the relevant statement unit as *SEMUNIT: negation unit*, enabling consistent interpretation and presentation while avoiding OWL-specific modelling artefacts.

**Conceptual grounding of negations**: The treatment of negations in the Semantic Units Framework is conceptually aligned with Peirce’s *existential relational graphs*^81,82^, where a *line of identity* ‘**―**’ denotes that ‘*something is A*’ (***―****A*) (Fig. 12g). The *line of identity* can be understood to represent an existential quantifier (Ǝ*x*). The interruption of this line with a circle enclosing *A* denotes negation, i.e., ‘*something is not A*’ (￢*A*). The resulting existential relational graphs are sufficiently general to represent full First-Order Logic with equality^82^, and conceptually mirror our use of the negation unit.

**Fig. 14:**
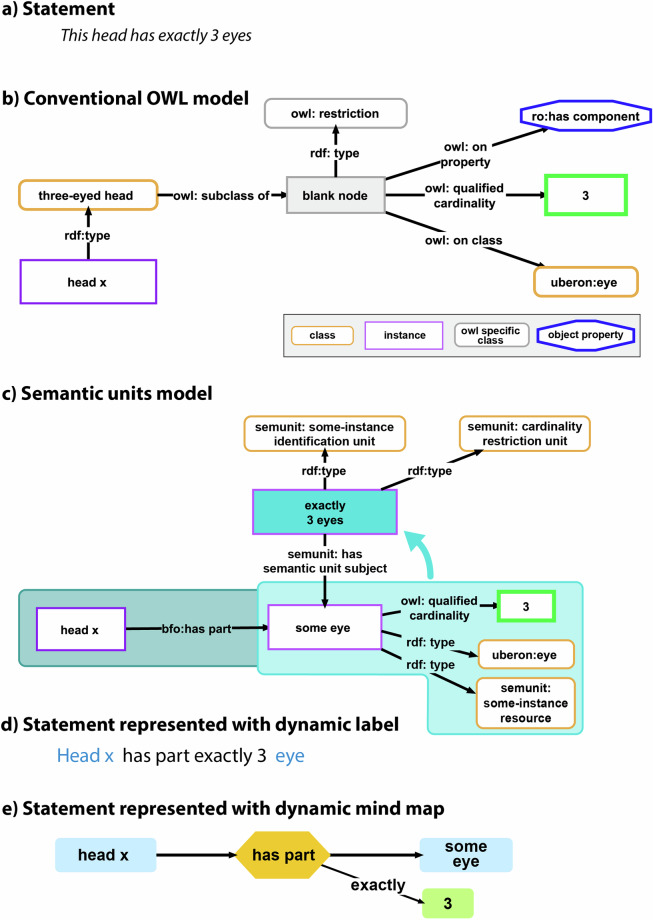
Representing cardinality restrictions within the Semantic Units Framework. (**a**) A human-readable statement that this head has exactly three eyes. The statement can be modelled in two different ways: (**b**) as an OWL TBox expression mapped to RDF. Note, how ‘has exactly three eyes’ is translated into a class expression with a cardinality restriction on the *has component* property (RO:0002180) and the class eye (UBERON:0000970) with a cardinality value of 3, thereby involving one blank node. (**c**) Alternatively, the statement can be represented as an instance-based ABox graph using two semantic units, one of which models the has-part relationship, with its content-graph shown in the dark-blue box. The other semantic unit (light-blue box) instantiates *SEMUNIT: assertional statement unit*, *SEMUNIT: some-instance identification unit*, and *SEMUNIT: cardinality restriction unit*. The semantic content of the two statement units can be displayed either as text through a dynamic label (**d**) or graphically as a dynamic graph (**e**). *For reasons of clarity, metadata for the semantic unit is not represented*.

**Cardinality restrictions units**: Cardinality constraints in OWL are expressed using qualified cardinality restrictions within class expressions, which are difficult to apply at the instance level and challenging to query or visualize (see Section '*Challenge: Representing negations and cardinality restrictions*'). The Semantic Units Framework instead introduces *cardinality restriction units*, which attach quantitative constraints directly to instance-quantified resources (Fig. 14).

For example, the statement “*This head has exactly three eyes*” is represented by a has-part statement unit linking the head to a some-instance resource of type eye (UBERON:0000970), where the corresponding identification unit specifies a qualified cardinality (Fig. 14c). This allows cardinality, ranges, and frequencies to be expressed as an instance-level quantitative constraint using float values combined with appropriate units (e.g., count unit (UO:0000189) or percent (UO:0000187)).

**Formal semantics and translation to OWL:** Although semantic-unit-aware reasoners are not yet available, negation units and cardinality restrictions can be translated into OWL axioms using logic-based translation patterns, as outlined in prior work^80^. Cardinality restriction units correspond to OWL cardinality constraints over constructed collections, while negation units suppress standard assertion patterns and instead generate complement-based axioms or negative property assertions.

Importantly, the Semantic Units Framework does not mandate a single logical interpretation strategy. Instead, its modular structure allows individual statement units to indicate the logical or rule-based framework under which their semantics should be interpreted. This enables a single knowledge graph to coherently integrate classical OWL semantics, non-monotonic reasoning patterns, and quantitative constraints without forcing all content into a single formalism.

By reifying negation and cardinality as explicit semantic units, this approach enhances both cognitive interoperability (CLEAR) and machine-actionable semantics (FAIR), offering an intermediate solution that balances usability with formal rigor. It simplifies querying and visual representation, aligns with philosophical models of negation (e.g., Peirce’s existential relational graphs), and opens pathways for new types of reasoning in hybrid graph environments.

##### Addressing query complexity and interrogation statements

To address the challenges of query complexity (Section '*Challenge: The complexity of querying FAIR knowledge graphs*' and the lack of explicit support for representing interrogative statements (Section '*Challenge: Representing interrogative statements*'), the Semantic Units Framework includes *question units* (briefly discussed in^5^). Question units are a category of semantic units designed to represent interrogative statements as first-class objects within knowledge graphs (comparable to^83,84^), facilitating more intuitive and accessible querying mechanisms. Existing semantic unit classes can be used as sources that can be rephrased into interrogative forms, allowing queries themselves to be documented, shared, and reasoned over within the graph.

**Fig. 15:**
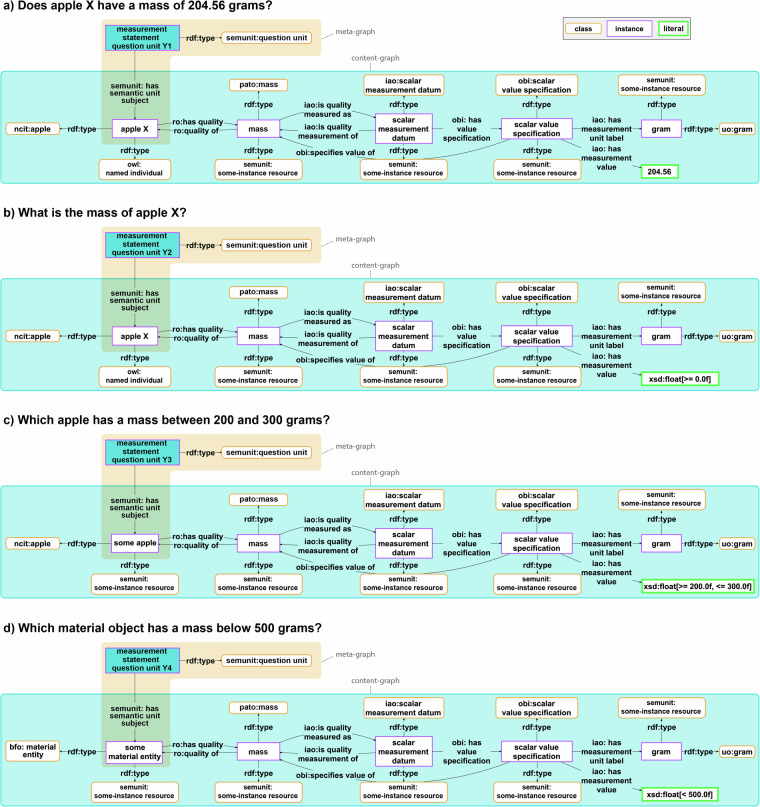
Question units. Four examples of question units. *For reasons of clarity, metadata for each semantic unit is not represented*.

A question unit can be derived from a source statement unit by copying its content-graph and classifying the resulting structure as a question unit. For example, the assertional statement “*Apple X has a mass of 204.56 grams*” (Fig. 4a) can be transformed into the question “*Does apple X have a mass of 204.56 grams?*” by reclassifying the copied content-graph as a question unit (Fig. 15a). When translated into an executable query, this question yields a Boolean result.

**Fig. 16:**
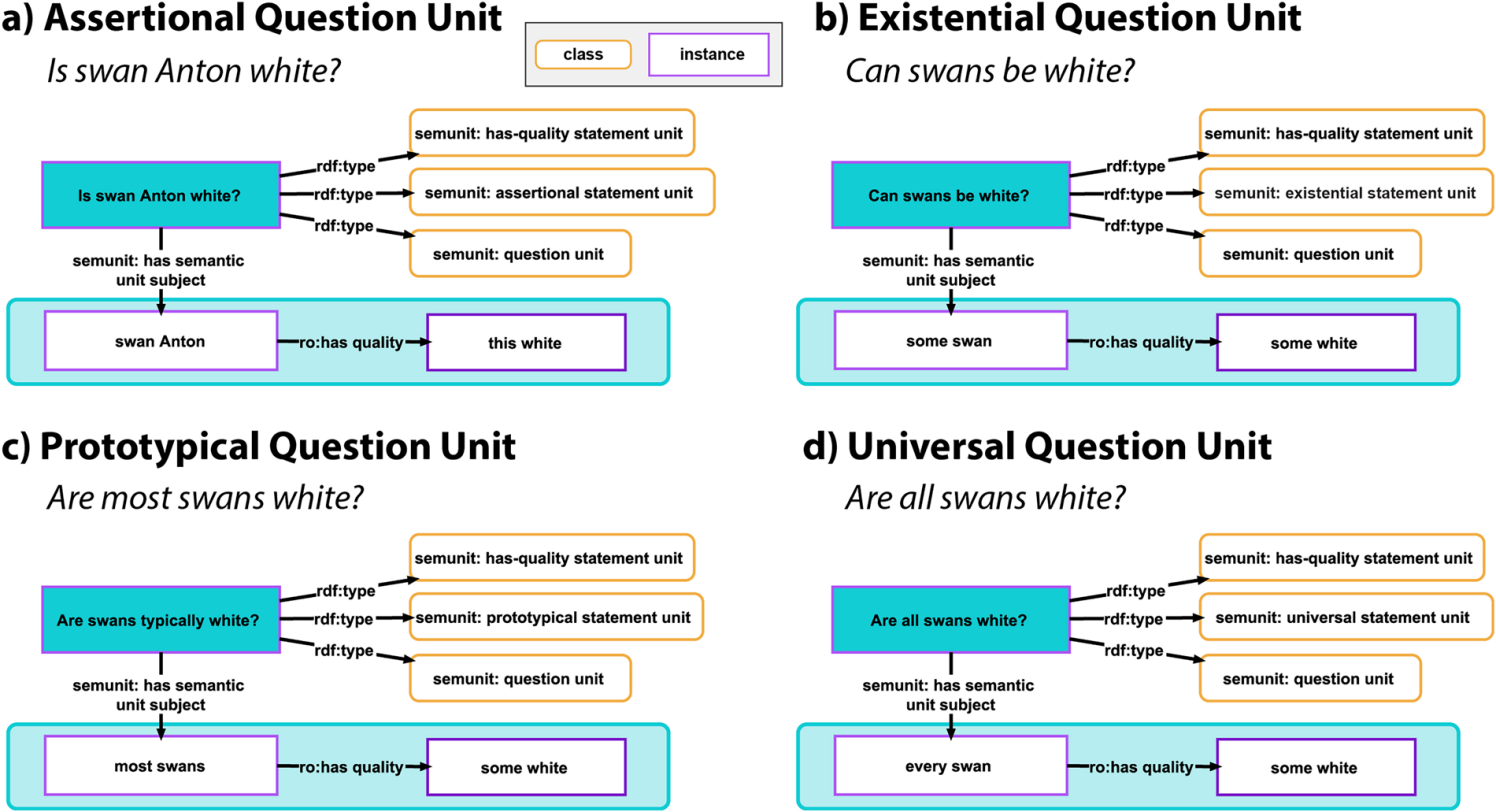
Representing interrogation statements as question units. Representing four types of interrogation statements using the statements from Fig. 8 as their sources. *For reasons of clarity, metadata for each semantic unit is not represented*.

More general interrogative forms are created by *underspecifying one or more slots* in the unit’s content-graph. Literal values can be underspecified by defining ranges (e.g., ‘*xsd:float[*> = *0.0* *f]*’), yielding questions such as “*What is the mass of apple X?*” (Fig. 15b). Similarly, subject and object resources can be underspecified by *replacing named individuals with instance-quantified resources*, enabling questions such as “*Which apple has a mass of 200 to 300 grams?*” (Fig. 15c). This mechanism generalizes across all statement unit categories, allowing assertional, existential, prototypical, and universal statements to serve as sources for interrogative representations. Depending on the type of source statement, corresponding classes of question units can be distinguished (Fig. 16).

The integration of question units with SHACL shapes facilitates the development of *query-builders* that can translate question units into executable queries. By utilizing SHACL shapes, we can derive input forms corresponding to the structure of the source statement unit, allowing users to construct queries without requiring familiarity with complex query languages (see Section '*Challenge: The complexity of querying FAIR knowledge graphs*'). The use of Boolean operators through *Boolean units* and the reusability of object resources as subject resources in another question unit facilitates the combination of multiple statement-based question units to form a more complex *compound question unit*. Depending on the Boolean operator, the following Boolean units can be distinguished: *Boolean AND*, *Boolean OR*, *Boolean XOR*, *Boolean NOT*, and *Boolean EQUAL unit*. It is imperative to note that all resources associated with a Boolean unit are interconnected through the unit’s designated Boolean operator.

By representing queries as semantic units, this approach reduces reliance on complex query languages, supports the documentation of competency questions or research questions directly within the knowledge infrastructure, and enables query construction, reuse, and explanation at the same semantic level as asserted knowledge. Question units thus provide a structured and cognitively accessible bridge between knowledge representation and interrogation in FAIR and CLEAR knowledge infrastructures.

##### Addressing standard views on data and knowledge

To address the challenge of representing standardized, topic-specific views as coherent knowledge objects (Section '*Challenge: Representing standardized, topic-specific views as coherent knowledge objects*'), the Semantic Units Framework includes *standard information units* as a specialized subcategory of compound units. A standard information unit groups together a curated set of statement and compound units into a semantically coherent, context-specific collection that represents a canonical view over a particular domain of knowledge and is itself represented as a first-class resource in the graph.

As a compound unit, each standard information unit is assigned a dedicated GUPRI, allowing its collection of semantic units to be referenced, annotated, cited, and reused as a single informational artefact. Unlike query-derived subgraphs/datasets or implicit groupings, standard information units explicitly represent such views within the infrastructure’s domain of discourse.

Which semantic units are included in a given standard information unit is determined by domain-specific expectations, standards, or workflows, and may reflect mandatory and optional content elements defined by external specifications. In many cases, a standard information unit is associated with a particular entity in the knowledge infrastructure (e.g., a material, product, organism, or person). This association is explicitly represented using the property *SEMUNIT: has associated standard information unit*, linking the described entity to the unit that provides its standardized description.

By formalizing standard views as compound units, the Semantic Units Framework enables:

- the identification and retrieval of semantically coherent information views as distinct semantic artefacts;
- the alignment of knowledge infrastructure content with domain-specific standards, formats, and schemata;
- the reuse, citation, and comparison of standardized views across systems and organizational boundaries;
- modular infrastructure construction supporting validation, provenance tracking, and versioning at the level of meaningful information collections.

Standard information units thus directly address the representational gap identified in Section '*Challenge: Representing standardized, topic-specific views as coherent knowledge objects*' by enabling standardized, topic-specific views to be modelled, exchanged, and reasoned about as first-class knowledge objects, while remaining fully integrated into a broader, heterogeneous knowledge infrastructure.

##### Addressing geo-indexed and temporally ordered data and knowledge

To address the challenge of representing spatio-temporal and sequentially contextualized knowledge (Section '*Challenge: Representing spatio-temporal and sequential information*'), the Semantic Units Framework extends both statement units and complex statement units to explicitly capture temporal, spatial, and ordering context while avoiding the fragmentation inherent in triple-based representations.

We therefore introduce the following three specialized assertional statement unit subtypes:

- *Time-index statement units* specify either a time point (e.g., a timestamp) or a time interval (e.g., a term of office or event duration).
- *Geo-index statement units* specify either geo-coordinates, administrative regions, or named locations.
- *Time-order statement units* locate a specific position within an ordered sequence, such as a position within a historical timeline, procedural workflow, or recurring event series.

These statement unit types can be used as *temporal*, *spatial*, and *sequential qualifiers* in combination with other statement units, forming *complex statement units*. The qualifiers are thereby represented as first-class semantic components of the complex statement unit, rather than being distributed across auxiliary triples or external metadata conventions.

Depending on the type of qualifying statement unit being used, we distinguish the following subtypes of complex statement units:

- *Time-indexed units* encapsulate statement units that are valid only within a specific temporal context and always include at least one time-index statement unit in an object position.
- *Geo-indexed units* contain information that is dependent on a location and therefore always include a geo-index statement unit in an object position.
- *Time-ordered units* cover statements that refer to a specific position within a sequential order and always include a time-order statement unit in an object position, enabling the construction of ordered timelines or procedural narratives.

**Fig. 17:**
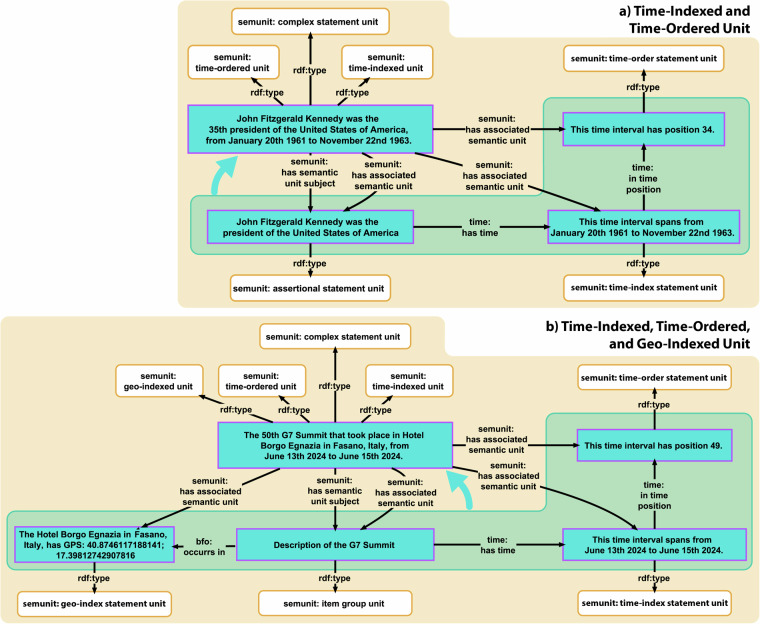
Representing time-ordered, time-indexed, and geo-indexed statements using time-ordered, time-indexed, and geo-indexed units. (**a**) Modelling the statements from Section '*Challenge: Representing spatio-temporal and sequential information*' about the presidency of John F. Kennedy as a complex statement unit. (**b**) Modelling of a G7 Summit with a specification of its duration, its position within the sequence of G7 Summits, and its location as a complex statement unit. *For reasons of clarity, metadata for each semantic unit is not represented*.

As an illustrative example, modelling the presidency of John F. Kennedy (Fig. 17a) involves:

- an assertional statement unit asserting that John F. Kennedy was president of the United States;
- a time-index statement unit specifying the term of office (1961–1963);
- a time-order statement unit indicating the position within the sequence of U.S. presidencies;
- and a complex statement unit that aggregates these statements into a cohesive, context-bound representation of the presidency, instantiating both a time-indexed and time-ordered unit.

Similarly, modelling the 50^th^ G7 Summit (Fig. 17b) combines:

- an item group unit describing the particular G7 Summit;
- a geo-index statement unit for the summit’s venue in Fasano, Italy;
- a time-index statement unit specifying the time interval during which the summit took place;
- a time-order statement unit for indicating the summit’s position within the historical sequence of G7 Summits;
- and a complex statement unit that aggregates these units into a cohesive information object, simultaneously instantiating a geo-indexed, time-indexed, and time-ordered unit.

By embedding temporal, spatial, and sequential context directly into a semantic unit structure, rather than attaching it indirectly through reification, Named Graphs, or ad hoc metadata, this approach preserves modularity, referential integrity, and semantic clarity. It enables precise querying, supports timeline and map-based visualizations, and facilitates reasoning over ordered processes and event sequences.

More broadly, this strategy addresses the fragmentation of meaning discussed in Section '*Foundational challenge: Fragmentation of meaning and loss of contextual coherence—the structural organization of meaning*' by ensuring that spatio-temporally qualified statements and statement collections are represented as coherent, first-class knowledge objects within the infrastructure.

Contemporary discussions on *context graphs* have highlighted the limitations of flat triple representations for associating events, places, time points, and temporal spans with assertions. By embedding spatio-temporal qualifiers as explicit components of complex statement units, the Semantic Units Framework provides a formal mechanism that answers the same structural need in a technology-agnostic, interoperable way.

##### Addressing directive statements

To address the challenge of representing directive statements introduced in Section '*Challenge: Representing directive statements*', the Semantic Units Framework includes the *directive unit* as a dedicated category of semantic unit designed to capture prescriptive content within knowledge infrastructures.

**Fig. 18:**
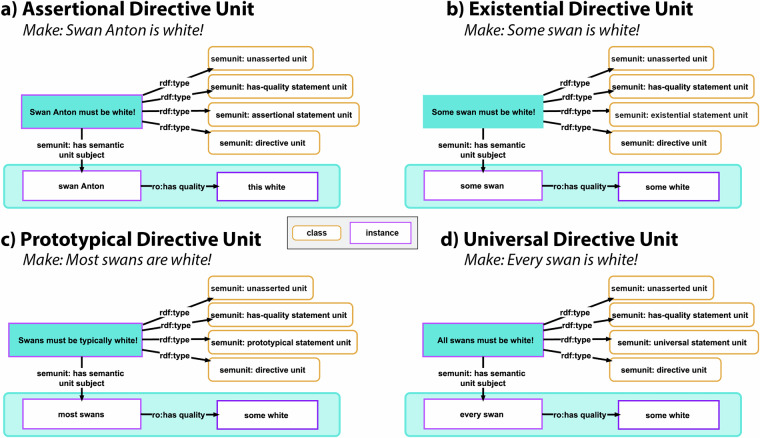
Directive units. Representing the four directive statements from Section '*Challenge: Representing directive statements*' as directive units. *For reasons of clarity, metadata for each semantic unit is not represented*.

A directive unit is created by classifying an existing statement unit additionally as an instance of a *SEMUNIT: directive unit* (Fig. 18), while at the same time indicating that it is non-asserted (see *unasserted unit* in Section '*Addressing non-asserted content*'). This allows the framework to reuse the full expressive structure of statement units while explicitly marking their non-descriptive, goal-oriented semantics.

In correspondence with the epistemic modality of the underlying statement, directive units are further distinguished into four subtypes: *assertional*, *existential*, *prototypical*, and *universal directive units*. This mirrors the classification of descriptive statement units and preserves distinctions in scope, generality, and intended applicability within prescriptive knowledge.

By retaining the original content-graph of the source statement, directive units ensure that the intended state of affairs is represented in a structurally identical way to descriptive statement units, while the additional directive typing signals that the statement expresses an obligation, intention, recommendation, or target state rather than an assertion of fact.

This design enables directive knowledge such as protocols, design goals, regulatory requirements, or workflow objectives to be represented in a formal, interoperable, and machine-actionable manner, alongside descriptive knowledge. It thereby supports reasoning over mixed epistemic content, where facts, expectations, and intended outcomes must coexist within a single knowledge infrastructure.

##### Addressing directive conditional statements

To address the challenge of representing directive conditional statements (Section '*Challenge: Representing directive conditional statements*'), the Semantic Units Framework comprises *directive conditional units* as structured complex statement units that explicitly link a condition (assertional or existential) to an action (directive). This builds on the more general notion of a *conditional unit*, which captures the logical relation between two statements.

**Conditional unit**: A conditional unit connects an *if-clause*, always represented as a non-asserted assertional or existential statement unit (see *unasserted unit* in Section '*Addressing non-asserted content*'), and a *then-clause*, which may be either a directive or a logical conclusion (see Section '*Addressing logical arguments*' below).

More complex conditions are supported using *Boolean units* (AND, OR, NOT, XOR, EQUAL), allowing multiple statement units to be combined into a single propositional block that can serve as the if- or then-clause.

**Fig. 19:**
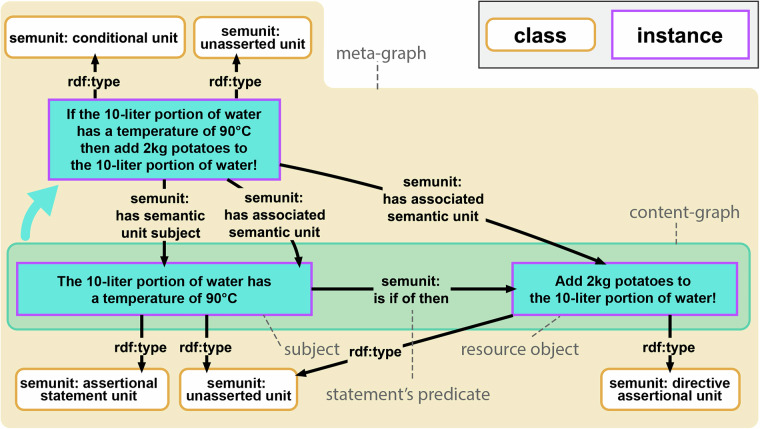
Directive conditional unit. Representing an if-then directive conditional statement as a directive conditional unit consisting of a non-asserted assertional statement unit as the if-clause and a non-asserted directive assertional statement unit as the then-clause. *For reasons of clarity, metadata for each semantic unit is not represented*.

**Directive conditional unit**: When the then-clause is a directive unit, the conditional unit becomes a *directive conditional unit* (Fig. 19). This is a *complex statement unit* that encodes both the truth-conditional semantics of its constituent statements and their procedural interdependency.

Technically, the directive conditional unit is constructed as follows:

- The *if-clause* (assertional or existential, non-asserted) is linked to the *then-clause* (directive, non-asserted) via the property *SEMUNIT:is if of then*.
- Both clauses are associated with the overarching conditional unit resource using *SEMUNIT:has associated semantic unit*.
- Human-interpretable labels can be dynamically generated by combining the *dynamic labels* of the assertional statement unit and the directive unit, prefixed with “*If*” and “*then*” respectively.

By formalizing directive conditionals in this way, knowledge infrastructures can represent dynamic, contingent procedures in a modular, queryable, and machine-interpretable manner, supporting action-based reasoning, automated workflows, and domain-specific instruction management.

##### Addressing logical arguments

To address the challenge of representing logical arguments in knowledge infrastructures (Section '*Challenge: Representing logical arguments*'), the Semantic Units Framework introduces *logical argument units* as a specialized form of conditional unit. Logical argument units make the structure of reasoning explicit by linking non-asserted statement units that function as premises and conclusions within a single, identifiable semantic object.

Each logical argument unit relates three distinct statement units^45^, each of which is non-asserted: A *case statement* (assertional statement or named-individual identification unit), a *rule statement* (universal, prototypical, or existential statement unit), and a *result statement* (assertional statement or named-individual identification unit).

Depending on the inferential direction, these statement units assume the roles of *premises* or *conclusions*. Logical argument units are thus conditional units that explicitly encode inferential structure, rather than leaving it implicit in infrastructure topology or external reasoning procedures.

**Fig. 20:**
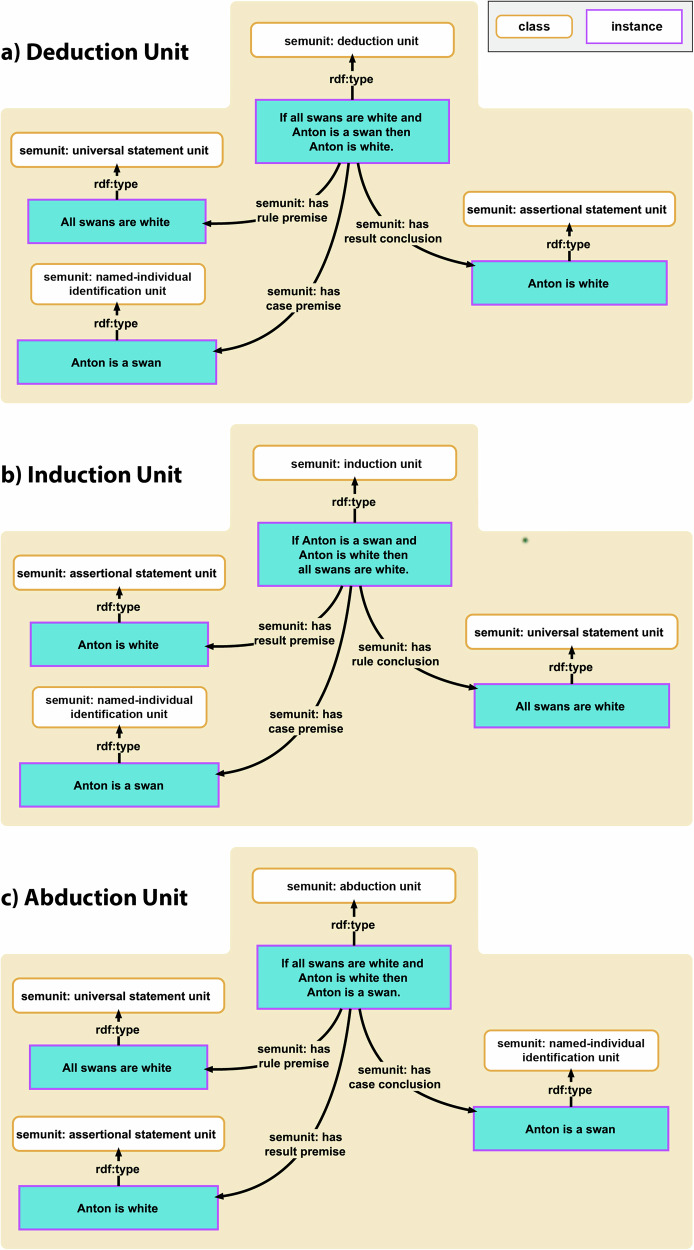
Logical argument units. Representing logical arguments as (**a**) a deduction unit, (**b**) an induction unit, and (**c**) an abduction unit. Not shown: every premise and conclusion statement unit is also an instance of *SEMUNIT:unasserted unit*. *For reasons of clarity, metadata for each semantic unit is not represented*.

**Types of logical argument units:** The framework supports three classical forms of logical reasoning by defining corresponding types of *logical argument units* (see Fig. 20):

1. **Deduction Unit: **Case + Rule ⇒ Result (Conclusion: assertional; modality: necessary)
2. **Induction Unit: **Case + Result ⇒ Rule (Conclusion: universal/prototypical/existential; modality: probable)
3. **Abduction Unit**: Result + Rule ⇒ Case (Conclusion: assertional; modality: possible)

These logical argument units associate their component statements using specialized subproperties of *SEMUNIT:has associated semantic unit* (Fig. 20). Where premises or conclusions are composite (e.g., conjunctions: “*X is white* AND *X has wings*”), Boolean units are used to combine multiple statement units into a single clause.

**Epistemic strength of inductive and abductive conclusions:** Inductive and abductive arguments differ from deduction in that their conclusions are not truth-preserving. The framework therefore supports explicit modelling of *epistemic strength*.

In inductive argument units, the inferred rule may vary in generality, from existential (“*Swans can be white*”) to prototypical (“*Most swans are white*”) to universal (“*All swans are white*”). While a single observation (e.g., “*Swan Anton is white*”) suffices to justify the existential claim (“*Swans can be white*”; a truth-preserving conclusion), it is insufficient for prototypical or universal claims (a not truth-preserving conclusion). These variants differ in evidential requirements and epistemic commitment, allowing the graph to distinguish cautious generalizations from stronger claims.

Similarly, abductive argument units may yield conclusions that are modelled either as *hypotheses* or as *presumed facts*, depending on the intended epistemic stance. This distinction avoids conflating plausible explanations with established knowledge and supports explicit tracking of conjectural reasoning (see also Section '*Addressing epistemic beliefs and disagreement*').

**Logical reasoning in practice**: Logical argument units enable the specification of *inference rules* within the knowledge infrastructure. These rules can be leveraged in reasoning systems to derive new conclusions, mirroring deductive, inductive, or abductive logic. For instance:

- If both premises in a deduction unit are present, a reasoner can assert the conclusion.
- Inductive reasoning rules can vary based on the epistemic strength of the inferred generalization.
- Abductive rules may produce tentative hypotheses that can be validated through further observation or cross-referenced data.

These reasoning mechanisms complement existing OWL reasoning but extend well beyond it, supporting *multi-premise, modality-sensitive inference* grounded in formal semantic structures.

The introduction of logical argument units marks a pivotal step toward empowering knowledge infrastructures with true logical expressivity. By capturing the structure and modality of deductive, inductive, and abductive reasoning, the Semantic Units Framework transcends static data and knowledge representation and moves toward *semantic reasoning as a first-class capability*. It also provides the foundational scaffolding for integrating argumentative structures into knowledge-centric AI, scientific publishing, hypothesis tracking, and intelligent agents.

When combined with the modelling of universal statements, directive conditionals, and Boolean units (as addressed in previous sections), this approach delivers a coherent, extensible paradigm for semantically rich and logically grounded knowledge representation.

#### Addressing general challenges of meaning, epistemics, and identity

This section demonstrates how the Semantic Units Framework addresses the general challenges outlined in Section '*From OWL limitations to general challenges of representing meaning, epistemics, and identity*', including non-asserted content, epistemic belief and disagreement, semantic fragmentation, and identity of granular entities, by providing explicit, modular units of meaning that preserve context and epistemic status in holistic knowledge infrastructures.

##### Addressing non-asserted content

The Semantic Units Framework addresses the challenge of non-asserted content by decoupling the *existence* of a statement in a knowledge infrastructure from its *epistemic endorsement*. Rather than treating statements as implicitly asserted facts, as is the case for RDF triples within OWL, the framework represents statements as first-class semantic objects whose epistemic status is made explicit.

Within this approach, each statement is instantiated as a statement unit, which can be further typed according to its epistemic role, for example as an *asserted unit* or an *unasserted unit*. This allows a knowledge infrastructure to represent propositions as objects of discourse without committing the infrastructure itself to their truth. The framework thereby separates the representation of a statement from its endorsement, independently of any beliefs, evaluations, or commitments that may later be associated with it.

For example, a claim such as “*Agent A asserts statement X*” is modelled as a complex statement unit by linking within its content-graph the agent to a statement unit that encodes the propositional content of *X*, while this statement unit remains explicitly unasserted by typing it as instance of *SEMUNIT:unasserted unit*. The knowledge infrastructure thereby represents the *act of assertion* without asserting the content of statement *X*. This separation enables the representation of reported, hypothetical, disputed, or speculative content without unintended truth propagation. It also allows the representation of directive, conditional directive, and logical arguments without necessarily asserting the statements these involve (see sections above).

In contrast to RDF reification, Named Graphs, or RDF-star, which treat statements primarily as technical constructs for annotation, semantic units treat statements as epistemic objects with explicit status. Existing approaches either lack formal mechanisms to distinguish asserted from non-asserted content or rely on conventions that remain external to the infrastructure’s semantics. As a result, they are prone to ambiguity regarding whether a statement is merely reported or endorsed.

By making epistemic status an explicit and queryable property of statement units, the Semantic Units Framework allows contradictory or competing claims to coexist within the same knowledge infrastructure at the representational level without inducing inconsistency. This aligns with the framework’s broader treatment of statements as modular semantic artefacts, enabling fine-grained attribution, comparison, and contextualization.

More generally, this design allows knowledge infrastructures to also function as *libraries of claims* rather than only flat *databases of truths*. Non-asserted content becomes a first-class modelling construct rather than an exceptional case, providing a principled solution to a long-standing representational limitation in RDF/OWL based knowledge representation.

##### Addressing epistemic beliefs and disagreement

Having established how statements can exist within a knowledge infrastructure without being asserted as true (Section '*Addressing non-asserted content*'), we now address how semantic units enable the explicit representation of epistemic belief, disagreement, and attribution among agents. Once statements are represented as first-class semantic objects with explicit epistemic status, they can themselves become objects of belief, rejection, or uncertainty.

Statements may thus be referenced, attributed to agents, compared, or evaluated independently of whether they are endorsed by the infrastructure. This separation between propositional content and epistemic attitude provides the foundation for representing disagreement, competing interpretations, and higher-order beliefs within a single coherent knowledge infrastructure.

To address epistemic beliefs and disagreement, the Semantic Units Framework includes *epistemic units*―a specialized class of complex statement units that explicitly associate a person or agent with a statement, thereby encoding that person’s *epistemic stance* regarding that statement.

The framework defines a taxonomy of epistemic units, comprising three primary categories, each representing a fundamental epistemic attitude:

1. *Positive epistemic units* model affirmation or belief in a statement (e.g., *Person A* believes “*This fruit is a pome fruit*”) (Fig. 21a).

**Fig. 21:**
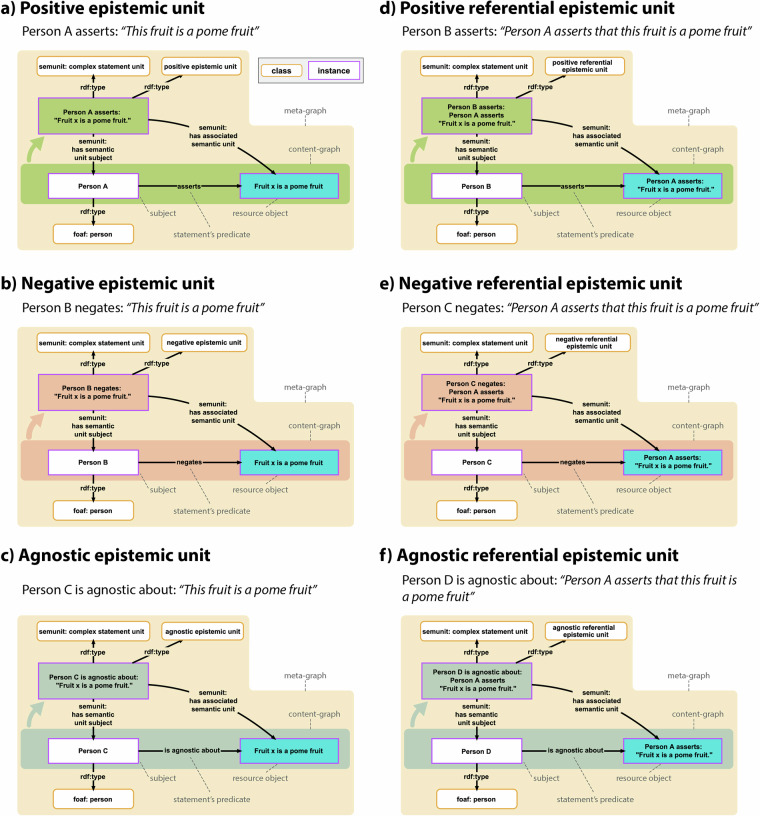
Representing epistemic beliefs and disagreement in the Semantic Units Framework. Six examples of different types of epistemic units. *For reasons of clarity, metadata for each semantic unit is not represented*.
2. *Negative epistemic units* represent disbelief or rejection of a statement (e.g., *Person B* rejects “*This fruit is a pome fruit*”) (Fig. 21b).
3. *Agnostic epistemic units* encode agnosticism or uncertainty regarding a statement (e.g., *Person C* neither affirms nor denies “*This fruit is a pome fruit*”) (Fig. 21c).

Each epistemic unit links an agent to the GUPRI of the relevant statement unit, which may be typed as unasserted unit or as asserted unit. This structure enables straightforward querying: retrieving all positive, negative, or agnostic epistemic units referencing a given statement provides a clear map of who agrees, disagrees, or is uncertain about that statement.

**Referential epistemic units: Modelling beliefs about beliefs**: Beyond direct epistemic stances, scientific discourse frequently involves *epistemic meta-attributions*, where an agent holds a belief regarding another agent’s belief. For example, *Person B* may hold the belief that *Person A* believes “*This fruit is a pome fruit*”. Such referential epistemic beliefs require a more expressive representational construct.

We model these using *referential epistemic units*, which are analogous to the primary epistemic units but differ in that their object positions are themselves epistemic units (Fig. 21d-f). Referential epistemic units can be:

1. *Positive referential epistemic units*: *Person B* asserts that *Person A* believes “*This fruit is a pome fruit*” (Fig. 21d).
2. *Negative referential epistemic units*: *Person C* denies that *Person A* believes “*This fruit is a pome fruit*” (Fig. 21e).
3. *Agnostic referential epistemic units*: *Person D* is uncertain about *Person A*’s belief in “*This fruit is a pome fruit*” (Fig. 21f).

This recursive structure enables rich modelling of *second-order epistemic attitudes* and facilitates capturing the full complexity of scientific debate, consensus formation, and disagreement.

**Extending epistemic modelling through logic programming:** While epistemic units are primarily a representational construct, they also provide a natural interface to formal reasoning frameworks. In particular, *logic programming* offers one possible way to operationalize reasoning over epistemic relations between statements^80^. Each epistemic unit, such as those shown in Fig. 21, is not only a standalone instance (an *epistemic statement unit*) but also serves as a referential anchor in a logic program. These statement units can be translated into OWL axioms via the transformation patterns described in previous work^80^. Moreover, by treating epistemic statement units as individuals within logic programs, relations between statements themselves, such as belief, contradiction, or support, can be explicitly modelled.

This dual-level modelling enables the use of *answer set programming*^85–89^ for expressing and reasoning over argument structures, for instance, to detect conflicts between beliefs, resolve inconsistencies, or generate alternative belief sets under different assumptions^90,91^. In this way, semantic units do not merely serve as a static knowledge representation mechanism but can also potentially participate in dynamic, rule-based reasoning environments, supporting applications such as scientific debate modelling, provenance analysis, and epistemic network visualization. *Note, however, that the use of answer set programming here is illustrative rather than prescriptive*.

##### Addressing fragmentation of meaning and loss of contextual coherence

The Semantic Units Framework addresses fragmentation of meaning by introducing explicit, persistent semantic units as the primary carriers of coherent meaning, rather than treating meaning as an emergent property of loosely connected triples. Instead of decomposing knowledge exclusively into minimal atomic assertions, such as individual triples in RDF, some of which by themselves do not make sense to a domain expert (cf. triple, highlighted in red, in Fig. 4b), the framework enables statements, situations, arguments, and other semantic constructs to be represented as bounded, identifiable, and sometimes nested semantic objects with internal structure. Consequently, information granularity reflects the complex granularity of the reference system it represents^70^.

At the core of this approach is the treatment of semantic units as *semantic wholes*: each unit defines a clear boundary around a coherent piece of meaning and explicitly links the statements, resources, and contextual elements that belong together. Rather than distributing meaning implicitly across an open-ended graph or table neighborhood, semantic units make semantic scope explicit by construction. This applies equally to simple propositions, complex n-ary relations, argument structures, and contextualized claims, as demonstrated in Section '*Addressing paradigmatic OWL expressivity limitations*'.

By modelling individual statements and collections of them as first-class objects with internal and potentially nested structure, the framework avoids the need to reconstruct meaning solely through graph or table traversal or through schema interpretation. A semantic unit explicitly encodes which components jointly constitute a meaningful whole, distinguishing core content from contextual, epistemic, or additional information. This substantially reduces the interpretative burden on both human user and computational systems, particularly in query results and downstream analytical tasks.

This strategy reflects general principles for managing complexity in other domains. In natural language, complex meanings are routinely encapsulated in single terms that function as *placeholders* for longer descriptions, and in biological systems, stable complexity emerges through nested, self-maintaining units such as organelles, cells, organs, and multicellular organisms. Semantic units similarly manage information complexity by organizing knowledge into granular, nested, and reusable semantic wholes (cf^70^).

Importantly, semantic units do not replace graph-based or tabular representations but reorganize them around modular semantic building blocks. Each semantic unit may be realized using various technical implementations (Section '*The Semantic Units Framework*'), yet its identity, scope, and coherence are defined at the semantic level rather than emerging accidentally from graph or table structure. This modularity enables semantic units to be composed, nested, or related without dissolving their internal coherence, supporting scalable knowledge infrastructure construction without semantic dilution.

This explicit modularization directly addresses concerns raised in recent industry and AI practice discussions around so-called *context graphs*, which highlight the difficulty of representing temporal validity, provenance, decision context, and conditional relevance in flat triple-based graphs. Rather than introducing ad hoc context layers, the Semantic Units Framework integrates contextual coherence into the representation itself, allowing contextual dimensions to be attached to semantic units as part of their internal structure. Semantic units thus shift contextual coherence from an emergent property of graph or table structure to an explicit design principle of knowledge representation.

As a result, knowledge infrastructures built on the Semantic Units Framework can preserve semantic wholeness while retaining the flexibility and formal rigor of graph-based representations. Meaning is no longer fragmented across arbitrary collections of triples but organized into identifiable, reusable, and contextually coherent semantic artefacts. This design supports FAIR and CLEAR Principles by making the boundaries of meaningful information units explicit, enhancing interpretability, reuse, and cognitive interoperability across diverse applications.

##### Semantic units as digital objects: Representational identity in holistic knowledge infrastructures

The Semantic Units Framework addresses the identity problem of granular entities by counteracting the fragmentation that destabilizes identity in triple- or table-based representations. As argued in the section above, when the semantic content of an entity is distributed across loosely connected statements without an explicit boundary, the question of what constitutes “the entity” becomes underspecified. For entities with rich internal granular structure, such as material entities, processes, directives, datasets, or observations, identity cannot be separated from the semantic structure that defines them.

To resolve this, the framework introduces *semantic units* as explicit, bounded carriers of meaning and, where appropriate, aligns the identity of such a unit with the identity of the entity it represents. Concretely, an item unit describing a granular entity, such as a particular specimen from a natural history collection, may be identified by a single GUPRI that simultaneously serves as (i) the identifier of the semantic unit desribing this specimen as a structured semantic artefact and (ii) the identifier of the entity instance itself, typed as specimen (OBI:0100051), within the knowledge infrastructure. The content-graphs or -table rows and cells associated with the semantic unit contain the internal semantic structure of the entity, including its attributes, relations, participants, temporal and spatial contexts, while the GUPRI functions as a stable reference to the entity as a coherent whole.

Under this approach, the semantic unit is not treated merely as a document *about* an entity, but as the entity’s digital manifestation within the knowledge infrastructure. This directly counteracts semantic fragmentation by establishing a single, explicit boundary within which both descriptive structure and contextual metadata jointly constitute the entity’s identity. This identity alignment is a deliberate, explicit, and optional modelling decision, not an implicit conflation. For many entities of interest in scientific and data-driven contexts, the entity does not exist in the knowledge infrastructure independently of its structured digital representation. Instead, its identity, scope, and meaning are constituted by the semantic content captured within the corresponding semantic unit.

In this sense, certain compound units may be understood as *descriptive semantic digital twins* of the entities they represent. Rather than serving merely as descriptive records, such units provide a structured, machine-interpretable manifestation of an entity whose identity, scope, and context are explicitly articulated within the knowledge infrastructure. This notion of a digital twin is used here in a minimal and semantic sense: the unit does not aim to replicate physical behavior or real-time dynamics, but to provide a coherent, authoritative representation of the entity as it exists within the digital knowledge infrastructure.

This design directly addresses long-standing identity ambiguity in RDF and OWL practice, including *document*-*thing* confusion and the overuse of identity predicates such as *OWL:sameAs* and *IAO:isAbout* (IAO:0000136) to compensate for unclear representational boundaries. By providing a first-class semantic unit with a clearly defined scope, the framework stabilizes identity assignments and reduces the need for auxiliary identifiers, parallel representations, or ad hoc alignment strategies. Metadata such as provenance, authorship, temporal validity, or trust indicators can be attached unambiguously to the entity’s GUPRI, while the entity itself can participate naturally in relations, reasoning, and aggregation.

Importantly, the framework does not mandate identity alignment in all cases. In domains where a strict separation between an entity and its description is required, as for example due to independent versioning, differing lifecycles, access control constraints, or external tooling requirements, the semantic unit may be modelled as a distinct representational artefact linked to the entity via explicit relations. By making identity alignment optional and explicit, the framework supports both holistic and more traditional modelling regimes within a unified semantic architecture.

In this way, the Semantic Units Framework operationalizes emerging ideas of holistic knowledge infrastructures by treating certain entities as semantically articulated digital objects that simultaneously serve as structural anchors and metadata anchors. This dual role aligns with FAIR Digital Object thinking and supports the CLEAR Principle’s emphasis on human-interpretable, contextually explorable data and knowledge structures. Rather than functioning as collections of loosely connected descriptions, knowledge infrastructures can thus represent granular entities as stable, coherent, and semantically grounded units of identity.

### Relation to FAIR digital objects and existing work

This section positions the Semantic Units Framework with respect to the concept of a FAIR Digital Object as well as prior approaches to modularization, statement annotation, and semantic grouping in knowledge graphs. The framework is not proposed as an alternative serialization or reasoning formalism, but as a conceptual and semantic architecture that complements existing technologies while addressing representational limitations identified in the literature and by practitioners.

#### Semantic units and FAIR digital objects

Semantic units were explicitly conceived as candidates for FAIR Digital Objects (FDOs) in the work that originally introduced the concept. In particular, it was argued that semantic units naturally satisfy the defining characteristics of FDOs by providing globally unique, persistent identifiers; rich, machine-actionable metadata; explicit typing; and well-defined internal structure^69^. A dedicated chapter in subsequent work further elaborates how semantic units can function as FAIR Digital Object units within research data infrastructures, including governance, provenance, and lifecycle considerations^5^.

In the Semantic Units Framework, a semantic unit is a first-class digital object whose identity, scope, and meaning are explicitly articulated. Compound units, statement units, and their specializations provide bounded, semantically coherent units that can be independently identified, cited, versioned, and reused. This aligns closely with the FDO vision of moving from loosely connected data fragments toward actionable, self-contained digital objects^92,93^.

Importantly, the framework does not prescribe a specific technical realization of FDOs. Instead, it defines a conceptual grammar for identifying what should count as a digital object in a knowledge infrastructure. Concrete implementations may use different container technologies, such as nanopublications^50–53^ or RO-Crates^94^, as long as they preserve the identity, boundaries, and semantics of the unit.

#### Relation to nanopublications and statement-level publication models

Nanopublications^50–53^ provide a well-established mechanism for publishing individual assertions together with provenance and publication metadata. They address the important problem of attribution, trust, and citation at the statement level and have proven valuable in scientific knowledge dissemination.

Semantic units are conceptually compatible with nanopublications, and this relationship was already discussed in the original semantic units publications. In particular, it was shown that nanopublications can serve as one possible implementation format for statement units or as a publication vehicle for semantic units more generally^5,69^. In this sense, nanopublications can be seen as a concrete instantiation of certain semantic unit types.

At the same time, the Semantic Units Framework goes beyond nanopublications in several respects. First, it generalizes the notion of a “statement” beyond atomic assertions to include universal, existential, prototypical, conditional, epistemic, and argumentative structures. Second, it provides an explicit taxonomy of unit types and roles, enabling statements, arguments, beliefs, and entities to be modelled uniformly as first-class semantic objects. Third, semantic units are not limited to assertion-centric publication but support unasserted content, disagreement, defaults, and non-monotonic reasoning, which fall outside the standard nanopublication model.

Thus, while nanopublications offer a valuable publication mechanism, semantic units provide a broader conceptual framework within which nanopublications can be situated, extended, or combined with other representational forms.

#### Relation to RDF reification, RDF-star, and Named Graphs

RDF reification, Named Graphs, and RDF-star all aim to enable statements about statements in the sense of triples about triples. However, these approaches primarily treat statements as individual triples and thus technical artefacts, rather than as epistemic objects with explicit semantic roles.

RDF reification^46^ is widely regarded as verbose and difficult to query^95^, while offering no principled way to prevent unintended entailments. Named Graphs^47^ provide a lightweight grouping mechanism but lack formal semantics for assertion, belief, or endorsement. RDF-star^48^ improves syntactic convenience but remains triple-centric and quoted triples are still grounded in the truth-conditional semantics of RDF and OWL.

The Semantic Units Framework addresses these limitations by shifting the location of assertions from triples to semantic units. Statements are represented as identifiable semantic objects whose epistemic status, whether asserted, unasserted, hypothetical, prototypical, or contested, is explicitly modelled. This enables knowledge infrastructures to represent claims, reports, beliefs, and disagreements without conflating representation with endorsement, a distinction that is not natively supported in RDF or OWL.

In this respect, semantic units do not compete with RDF-star or Named Graphs at the syntactic level, but operate at a higher semantic level, providing explicit modelling constructs that can be implemented using these technologies while avoiding their conceptual ambiguities.

#### Relation to other modularization and graph-based approaches

A variety of approaches have been proposed to mitigate fragmentation and support modular reasoning over RDF graphs, including kernel-based methods for RDF graphs^96^, rooted graph fragments and least common subsumers^97^, and query tree abstractions for user-oriented querying^98^. These approaches typically define modules implicitly, based on graph structure, similarity, or query behavior.

The Unit Graphs framework^99^ provides a conceptually related approach that explicitly distinguishes between semantically meaningful *units* (e.g., propositions or lexical entries) and their *types*. This framework formalizes units as first-class resources, allowing metadata, identity, and structural constraints to be attached at the level of units. While Unit Graphs emphasize type-instance distinction, they do not fully integrate the range of statement categories, epistemic status, and compositional structures that the Semantic Units Framework supports.

Semantic units extend these ideas by providing a deliberate, semantically grounded modularization that is compatible with multiple technical implementations (RDF/OWL, relational tables, JSON-based systems, etc.). A semantic unit is not an emergent fragment computed from a graph, but a deliberately modelled unit of meaning with a defined scope, identity, type, and epistemic context. This explicitness supports human interpretability, stable identity assignment, and principled attachment of metadata and provenance, enabling modularity across heterogeneous representations, technological implementations, and reasoning frameworks.

While kernel methods, rooted graphs, and Unit Graphs are valuable for analytics, similarity computation, or type-instance modelling, they do not directly address the epistemic, contextual, and identity-related challenges discussed in Sections '*Conceptual challenges in FAIR and CLEAR data and knowledge infrastructures*' and '*Addressing the challenges using the Semantic Units Framework*'. Semantic units are therefore complementary to such approaches as they provide stable, semantically coherent modules that can serve as input to graph analytics, learning, or query abstraction, while simultaneously maintaining formal integrity and cognitive interoperability.

## Discussion and Scope

This chapter discusses the broader implications, scope, and limitations of the Semantic Units Framework. Rather than introducing additional representational constructs, it reflects on what the framework contributes at a conceptual and architectural level, how it reshapes the organization of knowledge graphs and related knowledge infrastructures, and under which conditions it is intended to be applied.

### Taxonomy of semantic unit types

Over the previous Chapter, we introduced a comprehensive set of semantic unit types designed to address a broad range of representational challenges in knowledge infrastructures. These semantic units serve as the *foundational building blocks* for structuring and categorizing data and knowledge, ranging from simple assertional to complex logical and conditional constructs.

**Fig. 22:**
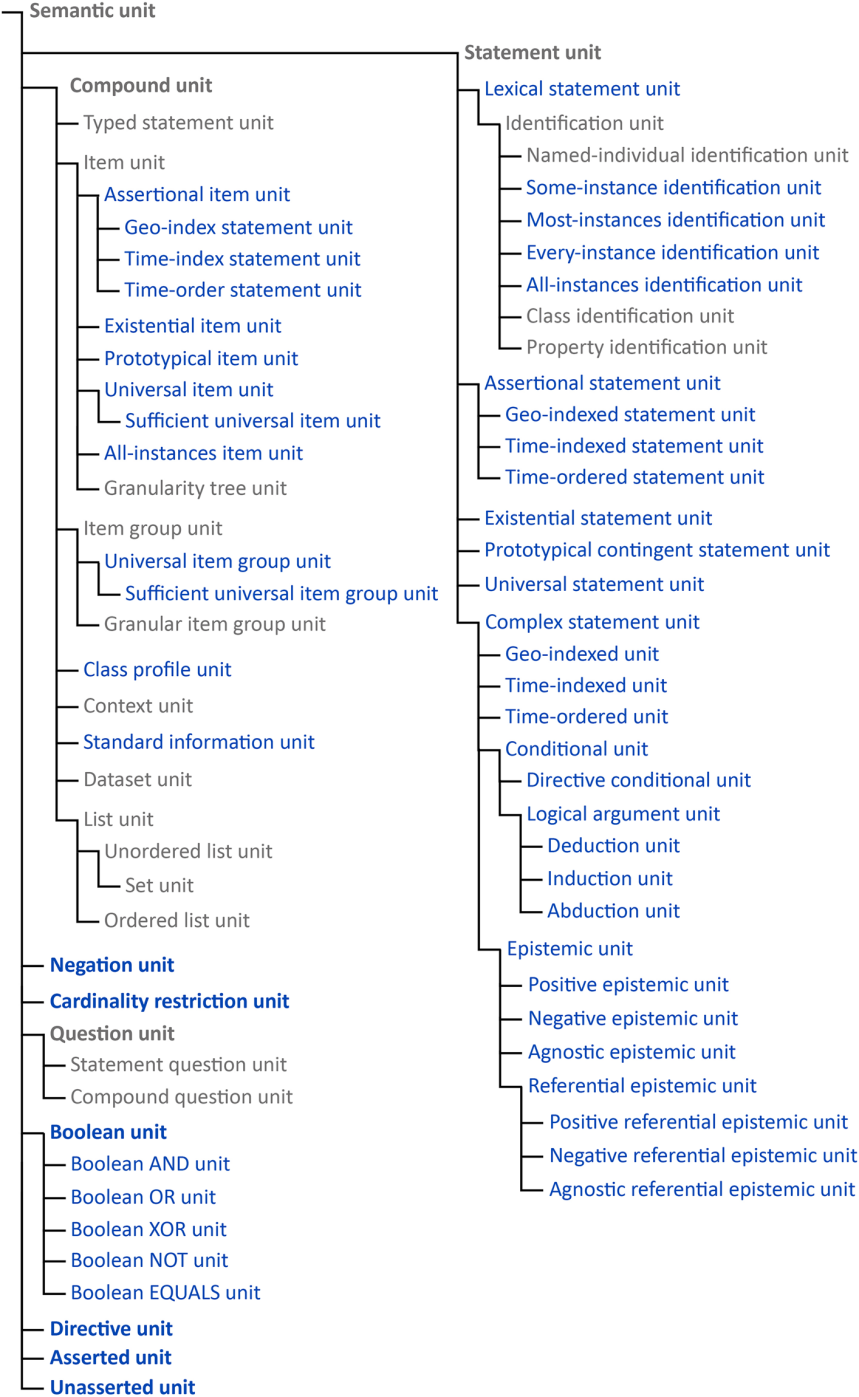
Classification of different categories of semantic units. All types of semantic units newly introduced in this paper are highlighted in blue. The remaining types (grey) have been introduced in previous work^5,69^.

Figure 22 presents the taxonomy of all semantic unit types developed in this and previous work. This taxonomy distinguishes:

- *Statement units* (e.g., named-individual identification units, assertional statement units),
- *Complex statement units* as a subclass of statement units (e.g., conditional units, directive units, epistemic units),
- *Compound units* used for designating semantically meaningful collections of statement and complex statement units (e.g., item units, class profile units, granularity tree units),
- and additional semantic units that serve the purpose to classify different semantic contents (e.g., Boolean units, negation units, cardinality restriction units, question units).

Each semantic unit class corresponds to a specific kind of information structure with a defined internal composition and external linking mechanism, enabling modular composition and explicit semantic scope. Each semantic unit type also explicitly states its logical or rule-based reasoning capabilities. This information plays a critical role in determining their compatibility with formal reasoning frameworks and provides the foundation for integrating semantic units with formal logical frameworks and OWL-extended knowledge graphs.

### Semantic modularization, unit granularity, and selective formalism in knowledge graphs

A central challenge in extending OWL-based knowledge graphs lies in reconciling the formal rigor of Description Logics with the epistemic diversity of real-world data and knowledge. OWL reasoners are designed for monotonic, truth-conditional inference, yet scientific and analytical practice routinely produces knowledge that exceeds these expressive limits, including probabilistic generalizations, defeasible claims, procedural instructions, and argumentative structures. In practice, this tension has led to two unsatisfactory alternatives: either such information is excluded from the knowledge graph altogether, or it is included without explicit indication of its logical or epistemic status, thereby obscuring what is logically valid, what is uncertain, and what is merely descriptive.

The Semantic Units Framework reframes this dilemma by rejecting the assumption that a single logical formalism must govern an entire knowledge infrastructure. Instead, it enables *semantic modularization*: the principled decomposition of a knowledge infrastructure into *logic-aware*, explicitly typed semantic units with well-defined representational roles. Each unit encapsulates a coherent piece of knowledge and, crucially, makes explicit whether and under which assumptions it is intended to support formal reasoning.

This organization supports what can be described as *selective formalism*. Knowledge that is compatible with Description Logics, such as assertional or universal statement units, can participate fully in OWL-based reasoning. Other forms of content, including prototypical generalizations, procedural directives, or abductive hypotheses, remain explicitly outside the scope of monotonic inference while still being first-class citizens of the representational layer of the knowledge infrastructure. Rather than weakening formal reasoning, this separation protects it by making reasoning boundaries explicit and epistemically transparent.

Semantic modularization also allows heterogeneous forms of knowledge to coexist without semantic conflation. Different semantic units may rely on different inferential assumptions, or on none at all, without requiring global logical consistency across the entire graph. This makes it possible to integrate descriptive data, generalizations, hypotheses, and instructions within a single infrastructure, while preserving clarity about what is asserted, what is inferred, and what is merely proposed or reported. This architectural separation offers several key benefits:

- *Transparent reasoning scope*: Consumers of the graph know exactly which inferences are logically grounded and which are not.
- *Mixed reasoning support*: The graph can simultaneously support multiple reasoning paradigms without conflation or inconsistency.
- *Safe extensibility*: Future logic types (e.g., temporal, default, fuzzy, or legal reasoning) can be integrated without redesigning the existing data and knowledge.

From a broader perspective, this approach aligns with emerging views of knowledge graphs as holistic yet structured knowledge infrastructures, rather than flat repositories of facts. By embedding epistemic and logical boundaries directly into the representational architecture, the Semantic Units Framework enables knowledge graphs to accommodate the diversity of scientific knowledge without forcing it into a single logical mold or relegating non-conforming content to informal annotations or external systems. Instead, the framework allows knowledge infrastructures to evolve into *multi-logic*, *heterogeneous reasoning environments*, where each part of the graph is epistemically transparent.

In this sense, semantic modularization is not merely a modelling convenience, but an architectural principle. It allows knowledge graphs to remain machine-actionable and logically sound where appropriate, while remaining expressive, transparent, and cognitively interpretable in the presence of epistemic diversity.

### On the generality of the Semantic Units Framework

Although the Semantic Units Framework has been illustrated primarily using RDF/OWL-based knowledge graphs, its scope is not restricted to any specific representation technology. As established earlier, semantic units are defined in this work as abstract semantic constructs rather than as graph-specific modelling devices. This section therefore clarifies the scope and boundary conditions under which the framework can be meaningfully applied across heterogeneous data and knowledge infrastructures.

At a conceptual level, the framework rests on a minimal assumption: information that is intended to be interpreted, evaluated, or reused must be expressible as statements. Semantic units formalize this assumption by treating statements as first-class, identifiable units of meaning that can be referenced, composed, and contextualized independently of their concrete technical realization. This abstraction allows the framework to function across infrastructures that differ widely in storage models, query mechanisms, and implementation details.

To support semantic units in a technology-independent manner, a data or knowledge infrastructure must satisfy a small set of structural conditions. In particular, it must support (i) the decomposition of content into bounded, semantically coherent units, (ii) persistent identification of these units via GUPRIs, (iii) explicit referencing between units via their GUPRIs and without content duplication, and (iv) the ability to associate each unit with a declared semantic type that specifies its interpretive role. These conditions are deliberately minimal and do not presuppose graph-based storage or RDF-specific constructs.

In this respect, the Semantic Units Framework is complementary to identifier-centric paradigms such as FAIR Digital Objects, which provide a format-neutral abstraction layer for digital artefacts. As discussed in earlier work^5^, semantic units can naturally function as FAIR Digital Object–level entities, regardless of whether their internal content is realized as RDF graphs, property graphs, tables, or other structured data formats. The framework thus operates as a semantic organization layer rather than as a replacement for existing data models.

Importantly, the framework does not prescribe how semantic units must be implemented, stored, or queried. Its contribution lies in providing a *conceptual grammar* for structuring information into semantically coherent, referencable units that preserve context, scope, and interpretability across infrastructures. In this sense, the generality of the Semantic Units Framework is a matter of architectural scope rather than technical ambition: it defines *what* should be treated as a unit of meaning, while leaving open *how* such units are realized in specific systems.

### Limitations and future directions

The Semantic Units Framework is intentionally presented as a conceptual and architectural contribution rather than as a concrete software system or executable reasoning framework. Its primary aim is to provide a principled conceptual framework and structural grammar for organizing data and knowledge into semantically meaningful, epistemically transparent units of meaning, independent of any specific implementation technology. As such, several limitations reflect deliberate design choices rather than shortcomings of the approach.

First, the framework does not prescribe a specific reasoning engine, inference strategy, or execution model. While many semantic unit types are compatible with established reasoning paradigms, such as Description Logics, rule-based systems, or logic programming, the framework itself remains agnostic with respect to how reasoning is performed. This separation is intentional as it allows semantic units to coexist with heterogeneous reasoning approaches without forcing all content into a single formalism. However, it also implies that practical reasoning behavior depends on external tooling and conventions, which must be specified by concrete implementations or application profiles.

Second, the framework does not define canonical serialization formats, performance guarantees, or storage optimizations. Semantic units can be realized in RDF/OWL graphs, labeled property graphs, FAIR Digital Object infrastructures, or non-graph-based data and knowledge systems, but the operational characteristics of these realizations, such as query performance, scalability, or update semantics, are outside the scope of this work. These aspects are best addressed by implementation-specific guidelines and empirical evaluation rather than by the core framework itself.

Third, while the framework introduces a rich taxonomy of semantic unit types, it does not mandate which unit types must be used in a given domain or application. Effective adoption therefore requires shared modelling conventions, domain-specific profiles, or community agreements regarding which semantic unit types and which corresponding data schemata and ontologies are appropriate for particular use cases. Without such coordination, semantic units risk being used inconsistently, reducing interoperability benefits. This limitation is inherent to any flexible semantic framework and underscores the importance of governance and standardization efforts alongside technical design.

Several directions for future work naturally follow from this foundation. One important avenue is the development of implementation profiles and validation mechanisms, for example through SHACL or similar constraint languages, to support consistent instantiation of semantic unit types. Another is the exploration of tooling that assists authors and data curators in constructing, inspecting, and navigating semantic units, thereby lowering the barrier of adoption. Further work may also investigate empirical aspects, such as cognitive benefits of unit-based representations and their contextual exploration for human users, or the impact of semantic modularization on downstream AI and analytics workflows.

More broadly, future research may examine how semantic units can support emerging paradigms such as digital twins, executable research objects, or context-aware AI systems, and how they integrate with evolving standards for FAIR Digital Objects, nanopublications, and holistic knowledge graphs. By remaining deliberately abstract, the Semantic Units Framework is designed to accommodate such developments rather than constrain them.

In this sense, the framework should be understood not as a finalized solution, but as a stable conceptual foundation for the next generation of semantically grounded FAIR and CLEAR data and knowledge infrastructures, one in which expressivity, logical consistency, semantic interoperability, epistemic transparency, and cognitive interoperability are treated as first-class architectural concerns.

### Concluding remarks

This work introduced the Semantic Units Framework as a principled approach for structuring data and knowledge infrastructures around semantically meaningful, identifiable units of representation. By treating statements and structured collections of statements as first-class semantic objects, each equipped with their own GUPRI, the framework implements *semantic modularization* by organizing knowledge infrastructures into bounded, logic-aware, and cognitively accessible units that can be independently referenced, annotated, queried, and interpreted.

Combined with instance-quantified resources (*some-instance*, *most-instances*, *every-instance*, and *all-instances*), semantic units substantially extend the expressive range of knowledge graphs beyond paradigmatic OWL usage. They enable explicit representation of assertional, existential, prototypical, universal, lexical, interrogative, directive, conditional, and negated statements without collapsing these distinctions into a single logical regime. In doing so, the framework addresses key expressivity challenges identified in Section '*OWL Expressivity as a paradigmatic challenge*', including the representation of existential and prototypical knowledge, ABox-level universal statements, complex class axioms, cardinality constraints, and natural-language-like querying.

Beyond expressivity, the framework directly responds to foundational challenges discussed in Section '*From OWL limitations to general challenges of representing meaning, epistemics, and identity*'. By decoupling the existence of a statement from its epistemic endorsement, semantic units enable the representation of non-asserted content as first-class knowledge objects. Epistemic units further allow beliefs, disagreement, and higher-order attributions to be modelled explicitly, without inducing logical inconsistency. Compound units and complex statement units counteract the fragmentation of meaning inherent in triple-based representations by providing explicit and often nested boundaries of semantic coherence. Finally, by optionally aligning the identity of semantic units with the identity of granular entities, the framework stabilizes representational identity for material entities, processes, experiments, datasets, and other structurally rich entities, supporting holistic and context-aware knowledge infrastructures.

A central contribution of the Semantic Units Framework lies in its support for selective formalism. Rather than forcing all knowledge into a single reasoning paradigm, semantic units make explicit which parts of a knowledge infrastructure are governed by which logical or epistemic assumptions, and which are not intended for formal reasoning at all. This protects formal reasoning where it applies, while preserving the ability to represent contingent, procedural, epistemic, or generalizing knowledge that exceeds the expressive limits of Description Logics.

Importantly, semantic units are defined as abstract semantic building blocks rather than as technology-specific modelling patterns. While demonstrated using RDF/OWL, the framework is inherently technology-agnostic and aligns naturally with FAIR Digital Object thinking. Its contribution is therefore architectural and conceptual by providing a shared *semantic grammar* for organizing knowledge across heterogeneous and potentially federated and distributed infrastructures, rather than a fixed implementation or software stack.

The enduring value of the Semantic Units Framework does not lie solely in enhancing reasoning capabilities, but in the fundamental representational insights that knowledge infrastructures must accommodate the full diversity of statements that matter in scientific communication and analytical practice, including those that cannot be fully formalized within a single logical system. Semantic units provide a way to document, interlink, and contextualize such knowledge in a structured but technologically flexible, transparent, and reusable manner. In this sense, the framework bridges human semantic understanding and machine-actionable structure, supporting both FAIR-aligned computation and CLEAR-aligned interpretability and usability.

## Data Availability

No data is available with this work.

## Code availability

No code was generated for this work.
